# Open-Source Multiparametric Optocardiography

**DOI:** 10.1038/s41598-018-36809-y

**Published:** 2019-01-24

**Authors:** Brianna Cathey, Sofian Obaid, Alexander M. Zolotarev, Roman A. Pryamonosov, Roman A. Syunyaev, Sharon A. George, Igor R. Efimov

**Affiliations:** 1 Department of Biomedical Engineering, George Washington University, Washington, DC 20052 USA; 2 Laboratory of Human Physiology, Moscow Institute of Physics and Technology, Moscow, Russia; 3 Institute of Personalized Medicine, Sechenov University, Moscow, Russia

## Abstract

Since the 1970s fluorescence imaging has become a leading tool in the discovery of mechanisms of cardiac function and arrhythmias. Gradual improvements in fluorescent probes and multi-camera technology have increased the power of optical mapping and made a major impact on the field of cardiac electrophysiology. Tandem-lens optical mapping systems facilitated simultaneous recording of multiple parameters characterizing cardiac function. However, high cost and technological complexity restricted its proliferation to the wider biological community. We present here, an open-source solution for multiple-camera tandem-lens optical systems for multiparametric mapping of transmembrane potential, intracellular calcium dynamics and other parameters in intact mouse hearts and in rat heart slices. This 3D-printable hardware and Matlab-based RHYTHM 1.2 analysis software are distributed under an MIT open-source license. Rapid prototyping permits the development of inexpensive, customized systems with broad functionality, allowing wider application of this technology outside biomedical engineering laboratories.

## Introduction

**Figure 1:**
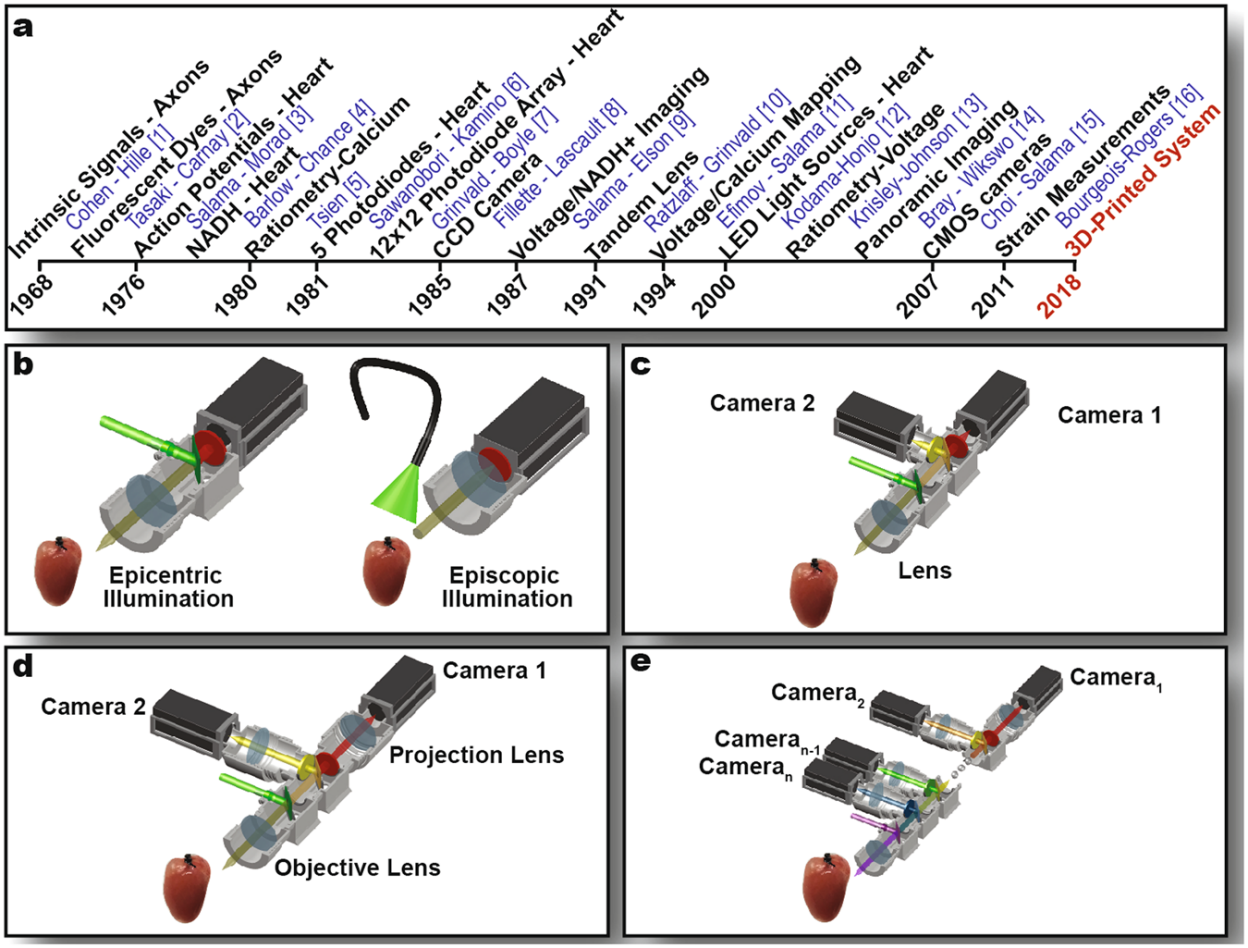
History of Optical Mapping. (**a**) Timeline of advances in optical mapping technology. (**b**–**e**) Optical mapping system setups with colored lines representing light path. Single-camera imaging is the simplest implementation. (**b**) Dual-camera imaging allows the simultaneous measurement of two parameters. (**c**) Tandem-lens arrangement (**d**) permits extension of the system for multi-parametric imaging (**e**).

Electrophysiology of excitable cells, such as cardiac myocytes and neurons, has been studied for more than a century using various electrode-based techniques to assess extracellular and transmembrane potentials, ionic currents and currents between two electrically coupled cells. However, these approaches are intrinsically limited in their ability to study excitable cells in relation to its surrounding multicellular environment with which it interacts, without compromising the spatial and temporal resolution of the techniques. Optical imaging was first developed to study axon electrophysiology as early as 1968 to overcome some of these limitations^1,2^ (Fig. 1a). Optical mapping utilizes either intrinsic fluorescent signals or fluorescent dyes that are sensitive to important physiological parameters such as NADH^+^, transmembrane potential, intracellular calcium concentration, etc., in order to study the related functions of the cell. Optical action potentials and NADH were first recorded from the heart in 1976^3,4^. The development of ratiometric techniques using BAPTA-based compounds as a fluorescent intracellular calcium indicator allowed for correction of artifacts from bleaching or changes in illumination intensity and focus^5^. Over the next decade, progress in optocardiography was due to the development of CCD cameras^6–8^, which allowed dramatic increase in spatial resolution. In 1987, two parameters – voltage and NADH^+^, were recorded from the same heart^9^. Later developments in this methodology included increasing the signal-to-noise ratio (SNR) and spatiotemporal resolution using tandem lens systems^10^. In 1994, the simultaneous recording of voltage and calcium from the same heart was reported^11^. Further advancements included the implementation of LED light sources^12^, ratiometric techniques for measuring voltage^13^, panoramic imaging systems^14^, and CMOS cameras^15^. A more recent development eliminates the requirement for uncoupling agents by using a motion tracking technique to subtract motion artifact, permitting the study of the relationship between electrical and mechanical activity in intact hearts^16^. Due to these developments, optocardiography has become a key tool in understanding the mechanisms of cardiac arrhythmias^17–19^.

The complexity of an optical mapping system is determined by the range of applications required. Single or multiple parameters can be recorded simultaneously by splitting emitted light using dichroic mirrors and using multiple photodetectors. In its simplest form, a fluorescent probe is excited and the emitted light is collected using a single lens and directed through an emission filter to a photodetector (Fig. 1b). The excitation light delivery could be episcopic or epicentric. Episcopic light source delivers light at an angle to the field of view, in contrast to precise illumination of just the field of view by directing the excitation light through the objective lens in the epicentric source. In a more sophisticated version of the system, two or more fluorescent dyes are perfused through the tissue and excited simultaneously. The emitted light is then collected by a lens, split into separate paths using a dichroic mirror, filtered, and directed to individual photodetectors (Fig. 1c). This system can be further expanded to record multiple parameters using a tandem lens approach (Fig. 1d). A tandem lens system implements two lenses per light path, with the fronts of the lenses facing each other to achieve an infinity-corrected light path between them. The infinity-corrected light path then allows the addition of multiple photodetectors to record several parameters simultaneously (Fig. 1e). The use of a tandem lens macroscope system has also demonstrated reduction in photobleaching and phototoxicity, increase in SNR, and production of brighter fluorescent images^10^.

Due to the complexity and high cost of optical mapping systems, this technique was initially limited to a few laboratories. Even though this technology became more easily available in the 1990s, the cost of implementation and experimentation still poses a significant limitation. The majority of the price is due to electronic equipment, including illumination sources and photodetectors^20^. Another cost to consider in optical mapping systems is the continued expense of conducting experiments that arise from the need for expensive dyes and chemicals, such as electromechanical uncouplers. Despite the high cost, a commercially bought system is specific to one application, making it difficult to adapt the system for newly developed protocols. Some of these cost and customizability concerns of optical mapping systems can be mitigated through 3D-design and printing. The components that support and position the acquisition and filtering equipment can be fully customized using 3D-printing, to accommodate and optimize the use of a wide range of specialized setups and tissue types. For example, tissue chambers can be 3D-printed at low cost in a size specific to the species of interest, which optimizes the volume of chambers and baths that house the tissue and thereby reduces the amount of dyes and other chemicals required. In other attempts to mitigate optical mapping costs, one study demonstrates low-cost CMOS cameras for panoramic imaging^20^, while others utilize a single detector for multiple parameters^21,22^. 3D-printing technology accommodates these new options for photodetector setups through customization. With open-sourced 3D-desings, researchers can design, build, or expand a system of necessary optomechanical equipment around a protocol, to optimize the application of costly electrical components. The system and its individual components can be easily built, scaled, and adjusted according to the type of tissue preparation and equipment being used.

While 3D-printing has previously been used to print lab equipment such as lab jacks, equipment stands and holders, optics equipment, tissue perfusion chambers, incubators, and guide electrical components for recording and stimulation, we present, for the first time, a fully 3D-printable optomechanical system for optical mapping to support the optical, perfusion and electrical components^23–30^. We also provide RHYTHM 1.2, an updated version of our previously published open-source optocardiography data analysis software, RHYTHM 1.0^31^, to analyze multiparametric optical mapping data, including voltage and calcium. On an open-source platform (https://github.com/optocardiography), we provide the designs of all system components as DWG and STL files for a 3D-printer and the Matlab code for data analysis. We demonstrate the use of our 3D-printed dual-camera tandem lens system by optically mapping voltage and calcium signals from intact mouse hearts and rat cardiac tissue slices.

## Results

This study implements and demonstrates the open-source optical mapping system illustrated in Fig. 1d. The system is comprised of three types of components: stage, optical, and perfusion components.

### Stage Components

**Figure 2:**
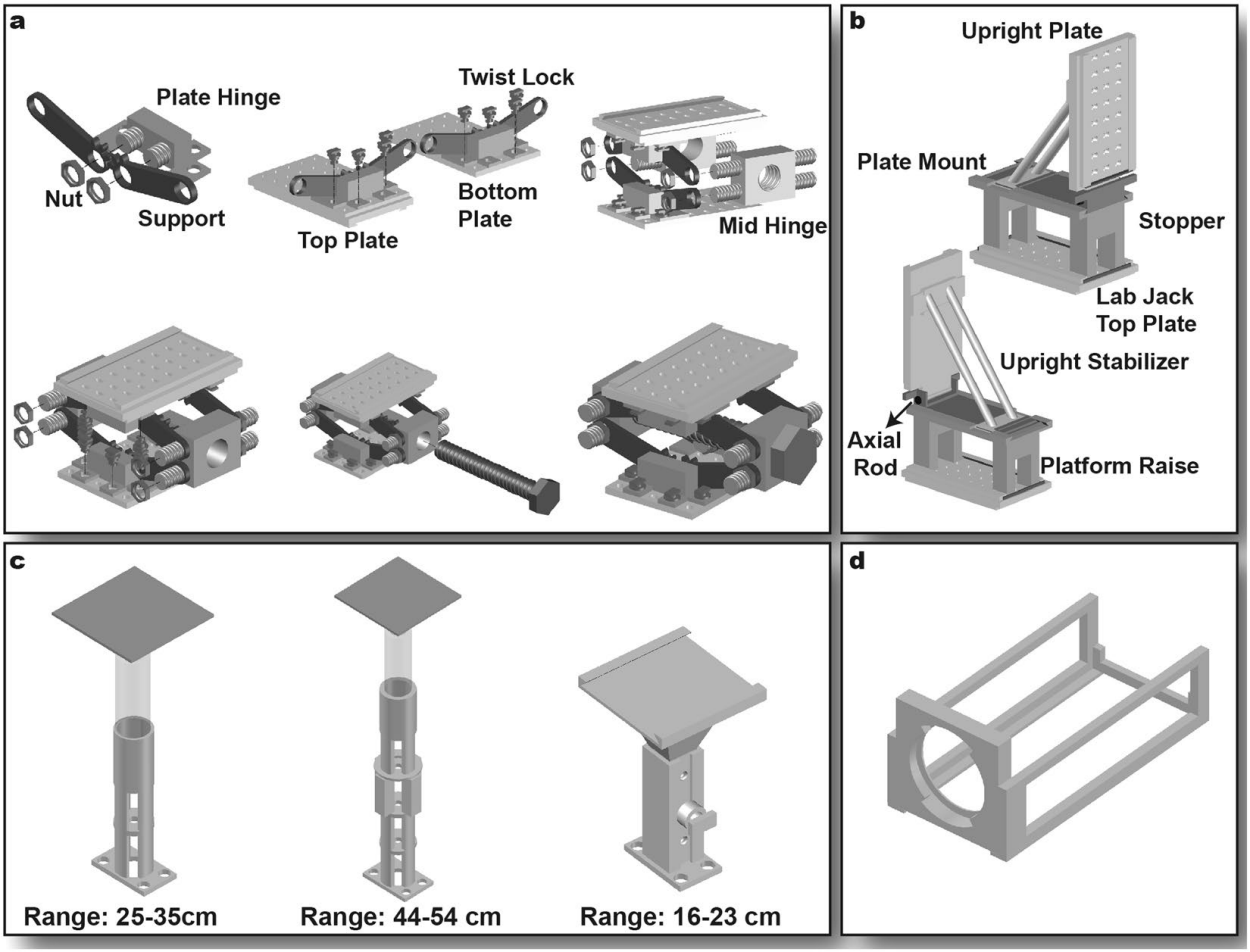
Stage Components. (**a**) Lab jack assembly. (**b**) Tilting platform for upright imaging mode. Emission and excitation filter cubes are mounted onto the upright plate. (**c**) Lifts. Two hydraulic camera lifts (left and middle) support the cameras in both system orientations. The upright bath lift (right) permits height adjustment of tissue preparation during upright imaging. (**d**) Camera cage secures cameras to projection lens sleeves.

The stage components include two lab jacks, a tilting platform, three hydraulic lifts, an upright bath lift, and two identical camera cages (Fig. 2). The purpose of these stage components is twofold: 1) to assemble and support the optical and perfusion components, and 2) to tilt the entire system from sideways to upright imaging mode, as required by experimental design.

The perfusion lab jack and optical lab jack that support the perfusion and optical components were printed with 27–34 cm and 15–23 cm height ranges, respectively, in order to align the optical and perfusion components during sideways as well as upright imaging. Both lab jacks were assembled in six steps (Fig. 2a), utilizing both a nut-and-bolt and a twist lock mechanism to secure pieces together. Featured mechanisms are illustrated in Supplementary Figs 1 and 2, accompanied by detailed assembly instructions. The lab jacks featured sliding rails on the top plate for securing perfusion and optical components. The lab jacks were able to support a load of up to 30 kg.

The tilting platform (Fig. 2b) allowed the optical components to be rotated 90**°** for upright imaging. The system was assembled in the sideways orientation, where the tilting platform was first secured onto the optical lab jack using sliding rails, followed by the optical components, which were secured onto the upright plate of the tilting platform in the same fashion. Next, the upright plate was rotated 90**°** from the horizontal position and the upright stabilizer was attached to the tilting platform to stabilize the optical components and prevent further tilting of the lab jack. Stoppers, shown in dark gray in Fig. 2b, provided additional support to the optical components. The tilting platform is further illustrated in Supplementary Figs 3 and 4, accompanied by detailed assembly instructions.

The upright bath lift (Fig. 2c, right), which consists of a vertically adjustable post and screw, allows for the positioning of a tissue bath within a 16–23 cm height range. This allows the tissue to be brought to the focal point of the objective lens in the upright orientation. The two cameras were supported by hydraulic lifts; two with a 25–35 cm height range for sideways imaging and one with 44–54 cm height range for upright orientation (Fig. 2c). The hydraulic lifts consisted of two 60 ml syringes (Cat# 13-689-8, Fisher Scientific) filled with water, separated by a 1-way stop cock (Cat# 120722, Radnoti). They could be positioned at any desired height by displacing the liquid in one syringe to the other. This additional support was required since the cameras were heavy and would otherwise not be in alignment with the rest of the system. The hydraulic lift positioning is further illustrated in Supplementary Fig. 5, accompanied by detailed instructions. While the hydraulic lifts supported the cameras in the vertical direction, a camera cage (Fig. 2d) secured each camera to the optical components using the twist lock mechanism. The hydraulic lifts were able to support a load of up to 15 kg.

### Optical Components

**Figure 3:**
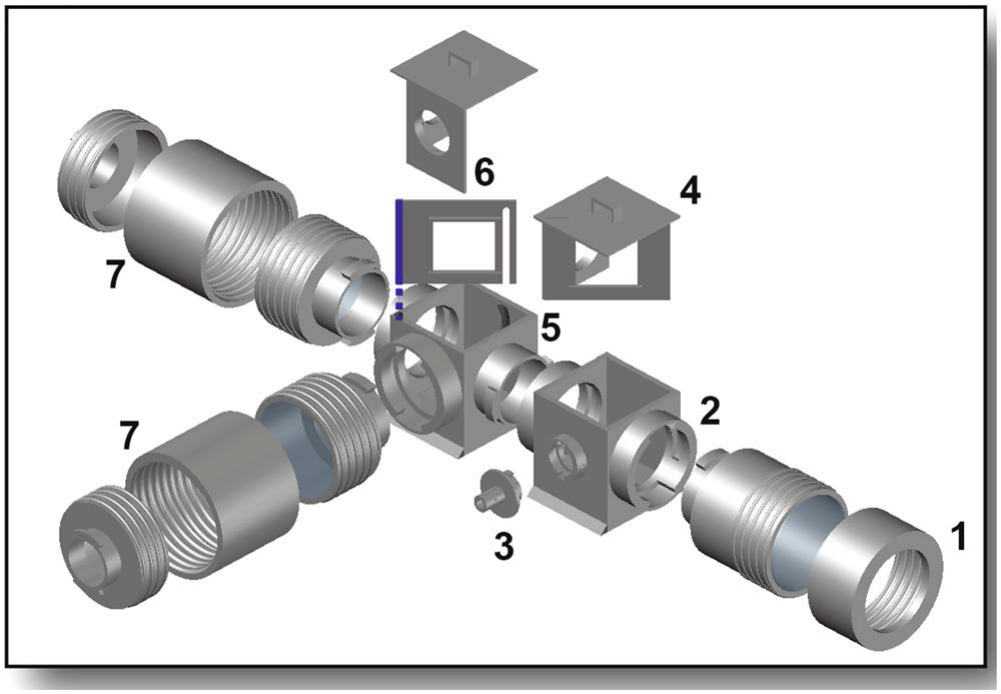
Optical Components. Expanded view of components housing filters, dichroic mirrors, and lenses (transparent gray). The objective lens sleeve (1) secures the objective lens to the excitation filter cube (2) that guides excitation light to the tissue preparation. The excitation light adaptor (3) secures the excitation light guide to the excitation filter cube. The stationary optics holder (4), placed in the excitation filter cube in the orientation shown, houses a dichroic mirror and features a circular emission filter holder for single-camera imaging. The emission filter cube (5) houses an adjustable wall (6) holding a second dichroic mirror that split the emitted light from the tissue preparation to two cameras (not shown). The projection lens sleeves (7) houses the projection lenses and secures one camera at the end of each.

The optical components consisted of the excitation and emission filter cubes, stationary and adjustable optics holders, objective and projection lens sleeves, and excitation light adaptor (expanded view in Fig. 3). These components housed the lenses, filters and dichroic mirrors and guided the excitation and emission light to and from the tissue preparation. Filters were fit into circular slots in the optics holder sets, while dichroic mirrors slid into rectangular slots in both optics holders.

The stationary optics holder was inserted into the excitation filter cube which positioned the dichroic mirror at 45° to the excitation light. The adjustable optics holder with the emission filters and dichroic mirror was inserted into the emission filter cube. The filter cubes were attached to each other using the twist lock mechanism and then to the tilting platform using the sliding rails and twist locks (Supplementary Fig. 1). The lenses were inserted into the objective and projection lens sleeves and were attached to the excitation and emission filter cubes, respectively. The projection lens sleeves feature a focal adjustor that was manually rotated to focus the cameras. The excitation light adaptor allowed the attachment of a light guide from the excitation light source to the excitation filter cube. Once the optical components were assembled and the cameras were attached to the projection lens sleeves, the images on the two cameras were aligned. This was done by adjusting the angle of the dichroic mirror inside the emission filter cube along the axis shown in blue in Fig. 3, which allows the dichroic mirror to be adjusted ±5° from the diagonal. The images were further aligned by finely adjusting the height of the cameras using the hydraulic lifts. The system achieved 99.07% spatial alignment, as illustrated in Supplementary Fig. 6, accompanied by detailed instructions and the alignment quantification method.

### Perfusion Components

**Figure 4:**
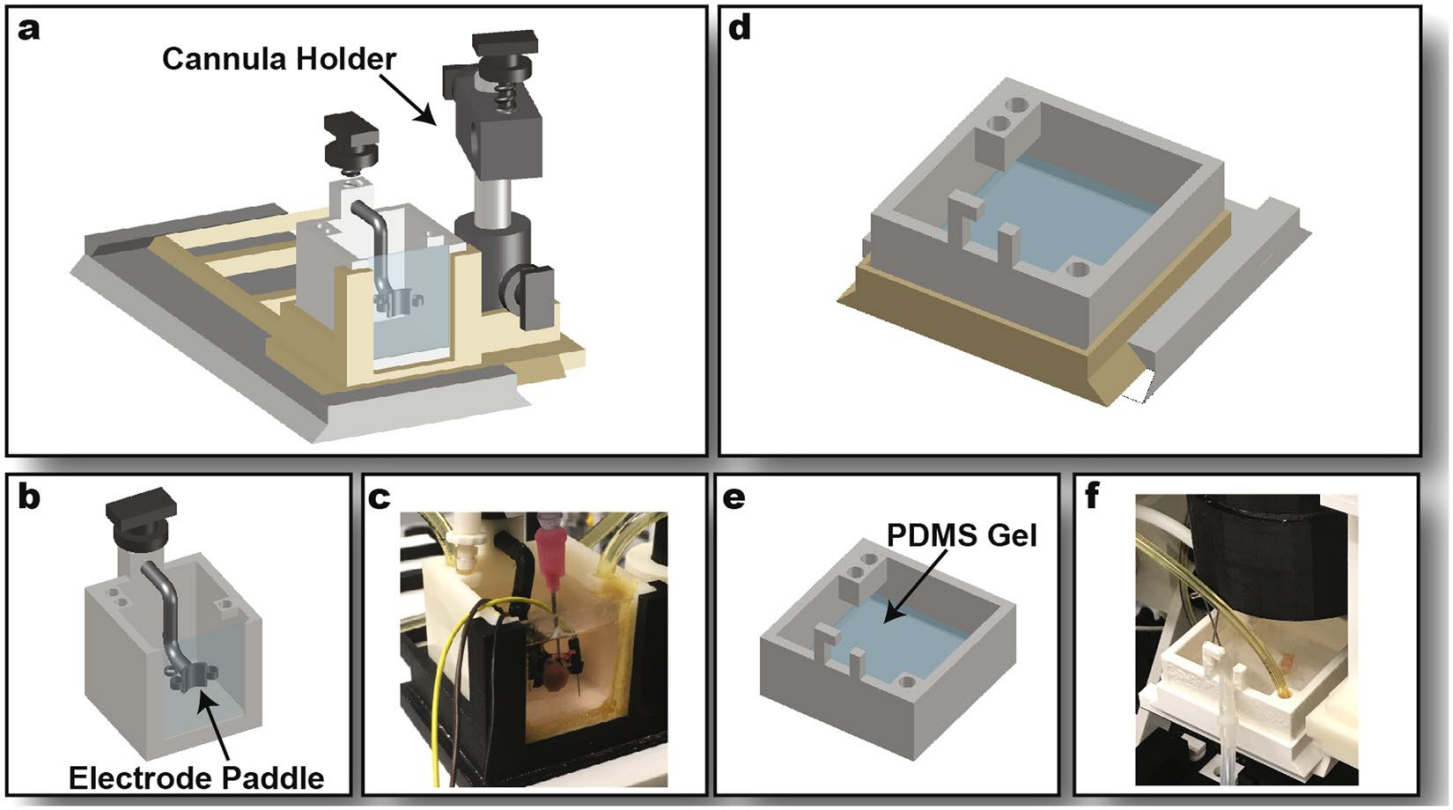
Perfusion Components. Sideways imaging components. The sideways bath stage (**a**) houses the sideways bath (**b**) and an adjacent cannula holder. Pseudo-ECG electrodes fit into the 3 slots of the electrode paddle that also stabilizes the heart against the optical window. (**c**) Image of printed sideways bath. Upright imaging components. The upright bath stage (**d**) houses the upright bath (**e**). PDMS gel secures insect pins holding tissue in place. (**f**) Image of upright bath.

The perfusion components include the sideways bath, the sideways bath stage, the upright bath and the upright bath stage (Fig. 4). The baths house the tissue preparations in temperature-controlled, oxygenated perfusate and the bath stages allow for xy-plane adjustment of the baths using sliding rails. The baths feature inlets and outlets to which silicone tubing is attached, that circulates the bath perfusate through a heat exchanger to keep the bath at an optimal temperature (37 °C). The inlets and outlets are placed at diagonally opposite ends of the bath to maintain uniform perfusate temperature and flow.

The sideways bath designed for a Langendorff-perfused mouse heart included an electrode paddle to stabilize the heart against the optical window and to hold the pseudo-ECG electrodes in place. Additionally, the sideways bath stage featured a cannula holder next to the tissue bath that held a cannula in place using a three-prong extension clamp (Cat# 05-769-6Q, Fisher Scientific). The sideways bath stage with its bath was mounted onto the perfusion lab jack.

In the upright bath designed for tissue slices or other flat preparations, PDMS gel in the inner bottom surface allowed for securing ECG electrodes and insect pins that hold the tissue preparation in place. The upright bath stage with its bath was mounted on the upright bath lift.

### System Assembly and Cost Comparison

**Figure 5:**
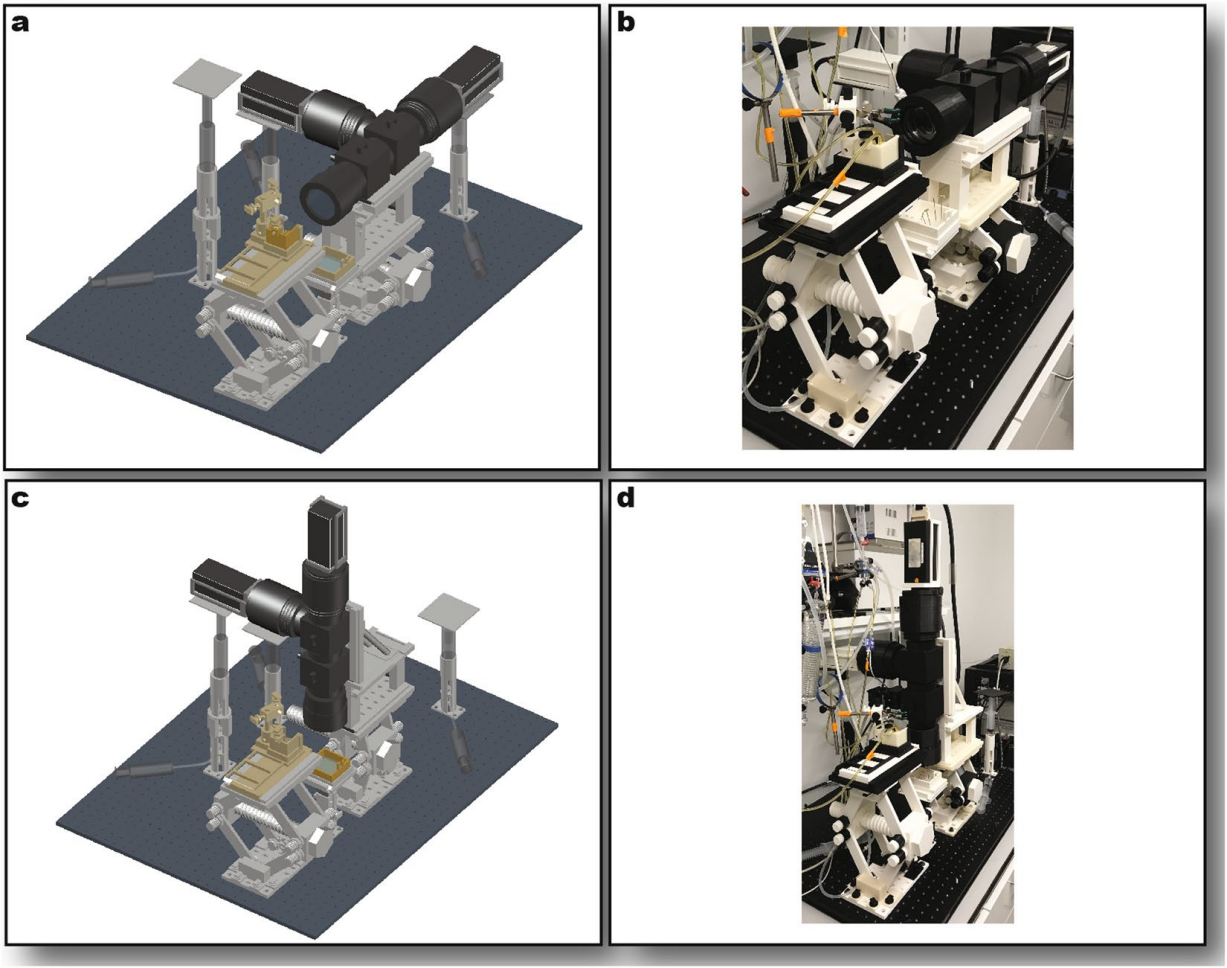
Full Optical System Assembly. Sideways imaging mode rendering (**a**) and photo (**b**). Upright imaging mode rendering (**c**) and photo (**d**). In the renderings, stage components are shown in light gray, optical components in dark gray, and perfusion components in gold.

The tandem lens optical mapping system was assembled as shown in Fig. 5. The system was designed to be capable of imaging in the sideways and upright orientation as illustrated in Fig. 5a–d. A manual of parts is provided in Supplementary Tables 1–3. The total cost of a fully 3D-printable optomechanical system was compared to commercially available equivalents and is detailed in Supplementary Tables 4–6. The commercial costs recorded were gathered from quotes provided by companies that sell optical mapping equipment and from other laboratory technology manufacturers. The total cost of 3D-printing customizable optomechanical parts necessary to support recording, illumination, and light filtering components is $1341 (Supplementary Table 5).

### Software

Custom Matlab software RHYTHM 1.2 was developed to analyze simultaneously collected voltage and calcium data. The open-source platform includes the software, a user manual, and sample data sets. The GUI provides the user the ability to condition signals, perform the subsequent analysis on a selected region of interest, and extract visuals. Voltage parameters calculated include action potential rise time (RT), action potential duration (APD), and conduction velocity (CV) using the Bayly method^32^. Calcium signal analyses include calcium transient RT, calcium transient duration (CaTD), and decay time constants (Tau). Four windows allow the user to view the optical recording, frame-by-frame, of up to four files independently and view their analysis maps. Statistical results are also displayed in the GUI for the analysis chosen.

### Functional Demonstration

**Figure 6:**
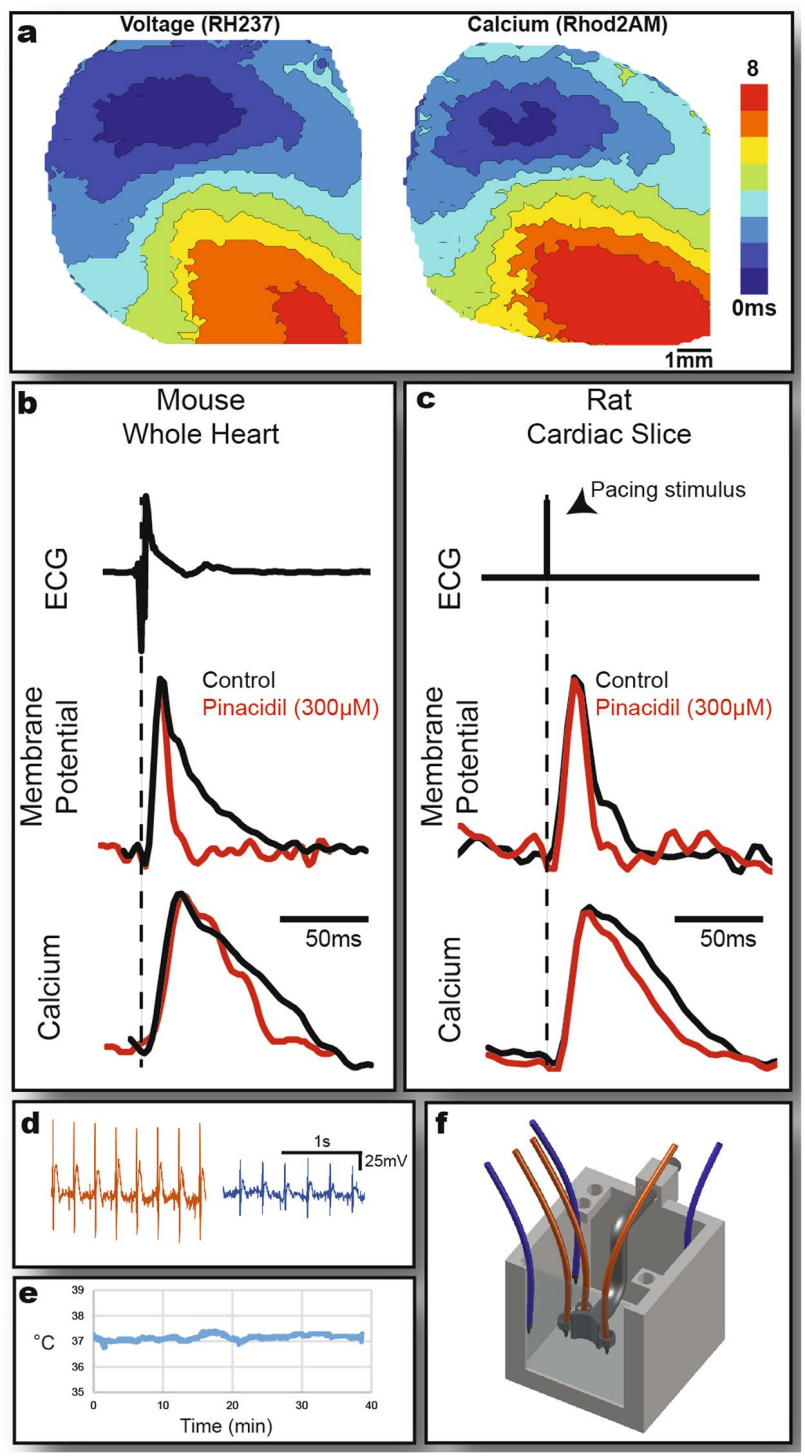
System Demonstration Data. (**a**) Activation maps of Langendorff-perfused whole mouse heart stained with voltage sensitive dye RH237 (left) and calcium sensitive dye Rhod2AM (right). Representative action potential and calcium transient recordings of whole mouse heart (**b**) and rat cardiac slice (**c**) during control Tyrode or 300 µM pinacidil treatment. (**d**) Comparison of pseudo-ECG traces using electrodes (+, −, gnd) placed in customized electrode paddle (orange) vs. traditional electrode placement (blue). The schematic (**f**) depicts electrode placement in each case. The graph (**e**) shows continuous temperature recording of a representative experiment.

Functional demonstration of the 3D-printed dual-camera tandem-lens system was performed using intact Langendorff-perfused mouse hearts in the sideways system orientation, and rat organotypic cardiac slices in the upright system orientation. Figure 6 and Table 1 display the experimental data. Representative voltage and calcium activation maps obtained from the mouse hearts are illustrated in Fig. 6a. Representative action potential (AP) and calcium transient (CT) traces from mouse hearts and a rat slice, during control conditions and after treatment with 300 μM pinacidil, are superimposed and shown in Fig. 6b,c, respectively. In whole mouse hearts, pinacidil (red trace) shortened APD relative to control (black trace), from 68.23 ± 1.67 ms to 44.04 ± 5.54 ms (p < 0.05) without significantly changing CaTD (Fig. 6b). Furthermore, pinacidil did not significantly alter other measured parameters in whole mouse hearts (Table 1). Supplementary Table 7 displays the mean parameter values obtained from both mice and rat.

**Table 1:** Voltage and Calcium Parameters for Whole Mouse Hearts Treated with Pinacidil.

	Parameter	Control	Pinacidil (300 μM)	p-value
Voltage	APD_80_ (ms)	68.23 ± 1.67^39^	44.04 ± 5.54	0.01
	RT (ms)	5.05 ± 0.03^40^	6.92 ± 0.72	0.19
	CV_T_ (m/s)	0.30 ± 0.02^41^	0.30 ± 0.04	0.96
	CV_L_ (m/s)	0.67 ± 0.03^41^	0.54 ± 0.09	0.32
	AR	2.24 ± 0.16^42^	1.79 ± 0.09	0.08
Calcium	CaTD_80_ (ms)	70.82 ± 5.66^39^	64.72 ± 5.03	0.51
	RT (ms)	13.98 ± 2.72^43^	18.054 ± 5.40	0.58
	Tau (s^−1^)	32.78 ± 12.59^44^	34.72 ± 15.15	0.93

Values are reported as mean ± standard error (n = 4). Voltage parameters include Action potential duration at 80% repolarization (APD80), rise time (RT), transverse conduction velocity (CV_T_), longitudinal conduction velocity (CV_L_), and anisotropic ratio (AR). Rise time was calculated as the time from 20% to 90% of maximum amplitude of upstroke of action potential. Anisotropic ratio was calculated as the ratio of CV_L_ to CV_T_. Calcium parameters include calcium transient duration at 80% repolarization (CaTD80), rise time (RT), and the calcium transient decay constant (Tau). 300 μM of pinacidil significantly reduced action potential duration (p = 0.01).

Pseudo-ECG traces (Fig. 6d) from a representative whole mouse heart were recorded by placing the electrodes in the custom slots on the sideways bath paddle (orange) and at the conventional location at the edges of the bath (blue) as illustrated in Fig. 6f. The close proximity to the heart and secure placement of the electrodes results in a higher quality signal with less noise and an approximately 35% increase in amplitude. The temperature of the bath was controlled and recorded throughout the experiments and maintained at 37.18 ± 0.16 °C (Fig. 6e).

## Discussion

Although several advancements in technology have improved optocardiography methodology and its scope over the last few decades, the cost associated with an optical mapping system is still a significant financial burden to many groups^20^. The advent of 3D-printing and its application in manufacturing components of experimental setups has opened a novel open-source avenue to design and produce the optomechanical components of an optical mapping system. Previous attempts at using 3D-printing in producing optomechanical components include replacement parts for a commercially available fluorescence microscope^33,34^, an LED brightness controller^28^, and a kinematic mount/platform to house mirrors^35^. Here, we describe for the first time, a fully functional, customizable 3D-printed system of optomechanical and perfusion components that support dual camera, tandem lens optical mapping studies. This approach also allows easy customization and rapid, inexpensive prototyping, providing both optical mappers and other investigators an alternative that mitigates the overall cost of optical mapping systems and catalyzes new study protocol development. The functioning of the system was demonstrated by optical mapping of whole mouse hearts and rat cardiac slice preparations, to simultaneously record voltage and calcium signals. Our custom designed ECG electrode holder improved the quality of the recorded pseudo-ECG traces. Additionally, we present here an updated optical mapping data analysis software, RHYTHM 1.2. While a previously released version of this software allowed for analysis of action potential duration, generation of activation and phase maps, RHYTHM 1.2 analyzes several additional parameters of voltage and calcium recording. Overall, a beginner or experienced cardiac electrophysiologist can easily replicate this open-source optocardiography system for research or teaching use without the need for manufacturing or software development competencies.

### System Features

The primary mechanisms for assembly of the system components were twist locks and sliding rails, both of which provided stability to the system. The twist locks permit a 90° rotation for locking. The only exception are the twist locks on the camera cages, which permit 360**°** rotation to provide the user with freedom in image orientation. The sliding rails allow for linear adjustments of the components in two dimensions. Both lab jacks and the tilting platform feature a grid of the twist locks and a sliding rail system along their lengths, to allow linear adjustment of both optical and perfusion components and to align and stabilize them. Furthermore, the assembly mechanisms of various components of the system are compatible such that each major component can be included or excluded depending on the application, and many parts can be modified in size or shape while retaining system compatibility. For example, the lens sleeves can be modified for different lenses, the tilting platform can be excluded in a system that does not require upright imaging orientation, an additional emission filter cube can be included to measure an additional parameter, and the stationary optics holder can house a filter for single-camera imaging. Furthermore, the individual components of the system can be scaled or easily modified to match other applications that vary in species of study and optics.

Multi-parametric optocardiography is an important tool in studies of the mechanisms of cardiac physiology. For multi-parametric imaging, spatial accuracy when splitting signals to multiple photodetectors using a dichroic mirror is essential. The design of the emission filter cube achieves this by allowing the dichroic mirror angle to be finely adjusted and then secured in place using 3D-printed clips. The hydraulic lifts also contributed to spatial alignment by allowing fine adjustment of the camera height.

For whole-heart studies, a high quality pseudo-ECG was recorded using the customized electrode paddle, which secured the electrodes in place to maintain polarity and close proximity to the tissue for increased signal quality. The curved surface of the paddle stabilized the heart against the optical window to create a flat imaging surface. A similar design goal has previously been achieved for a Langendorff-perfused mouse heart chamber, however this design featured a tissue restrainer device with a separate electrode holder attached to the chamber using screws^25^. While both of these chamber designs optimize placement of the heart and the electrodes, our new design achieves heart position stabilization and electrode placement by the paddle alone, which requires far less plastic and is more compact, a necessity for smaller tissue baths such as those for mouse hearts.

The waterproofing method used for the tissue chambers is effective for sealing off pores in ABS plastic, however if a different model material is desired, waterproofing measures may need to be modified according to material properties.

### Off-the-shelf (OTS) Components

One previous study in macroscopic imaging applications demonstrates an optical mapping system comprised entirely of OTS components^30^. Another study reported that the cost and complexity of fluorescent microscopy was effectively reduced by the assembly of a microscope that includes OTS components, including a dichroic mirror, an excitation filter, a barrier filter, a microscope, and a flashlight^34^. Yet another study presents an electrochemical-scanning probe microscope (EC-SPM) that uses 3D-printed stage platform and electrode holder, metal rods and an electronic stepper motor to produce motor-controlled linear stages for fine adjustment^28^. In these systems, 3D-printed parts are essential in two ways: they have a primary function within the system and secondarily, their customizability permits optimal incorporation of any OTS equipment. In all studies, simple OTS parts increase the range of application of low-cost 3D-printed components to make an integrated, customized system.

The system presented here incorporated several cost-efficient OTS components which improved functionality and usability of the system. The plastic syringes featured in the hydraulic lifts easily and precisely adjust the camera height and takes up little space on the setup. Three-prong extension clamps fit into the cannula holder and directly held the cannula and pacing electrodes in the sideways orientation. An optical window was used in the sideways bath to allow imaging of hanging heart preparations. Finally, PDMS gel was used to layer the inner bottom surface of the upright bath to secure tissue and ECG electrodes in place.

### Rhythm 1.2

The custom Matlab software, RHYTHM 1.2, expands upon a previous version of optical mapping data analysis software that we developed and published, RHYTHM 1.0^31^. The updates in this version of the software include analysis of voltage and calcium signals obtained using our dual camera system. In addition to the signal conditioning functions of RHYTHM 1.0, RHYTHM 1.2 is equipped to analyze and measure action potential durations, rise times and conduction velocities from voltage recordings, as well as calcium transient durations, rise time, and decay rate constants from calcium recordings. Software use was demonstrated by measuring each of the parameters listed above from both mouse hearts and rat cardiac slices. A detailed user manual and sample data sets are provided with the software on the open-source platform for other researchers to utilize or modify the software.

### System Demonstration

Dual imaging of voltage and calcium signals from whole mouse hearts and rat slices demonstrate effective functioning of the 3D-printed optical mapping system. Optical recordings obtained before and after administration of pinacidil, an adenosine triphosphate-sensitive potassium-channel opener, revealed APD shortening in whole mouse hearts, as previously reported^36–38^. Additionally, differences in other analyzed parameters in whole mouse hearts were not measurable between control and pinacidil treatment. In using a single rat heart slice preparation, we only demonstrate that the system can be used for physiological studies of different types of preparations. All values reported in Table 1 are comparable to previously published mouse data (references indicated in Table 1)^39–44^. Differences between these previously reported values are attributable to differences in protocol, including dyes, data acquisition method, and data analysis methods. Due to lack of previously published data from optical mapping of rat cardiac slices using similar optical mapping methods, there is limited ability to compare the parameters we report. More studies must be conducted to determine precise values for physiological parameters in rat cardiac slices, however we demonstrate that this optocardiography system can be used to do so.

### License

Hardware design was completed using a free academic license of AutoCAD. The Matlab code was written using a free academic license of Matlab 2017. The hardware designs and Matlab software are released with this publication under the MIT open-source software license.

## Limitations

While the 3D-printed system decreases cost and increases system functionality through customization, the production of the system requires access to an industrial-grade printer with a minimum resolution of 0.01in (0.254 mm). Most universities have in-house 3D-printing facilities, however prints can be outsourced to one of several commercial vendors including The UPS Store and FedEX. At this resolution, there is some variability present between replicates, which can alter the ease of fit between pieces. In this case, sand paper can be used to achieve the desired tightness of fit. The system also requires longer assembly time due to the number of parts and the use of OTS components. However, the additional assembly time is less than the time one is likely to wait for commercially ordered parts to be delivered. The modification of the open-source designs also requires 3D-design skills.

For effective dual mapping, the dyes chosen should have non-overlapping spectra. In this study we chose RH237 for membrane potential imaging and Rhod2-AM for calcium imaging^45^. RH237, though ideal for this application, yields weaker fluorescence intensity. This results in noisier signals especially in the thinner rat slice preparations. This can be further developed using better fluorescent markers with suitable spectra.

## Methods

All experimental protocols were approved by the Institutional Animal Care and Use Committee at The George Washington University and conform to the NIH Guide for the Care and Usage of Laboratory animals.

### Printing and Design of Hardware

Academic version of AutoCAD 2017 was used for the 3D design of all system components. To achieve optimal fits between attached pieces, distances ranging from 0.1–0.5 mm were left between adjacent surfaces depending on total surface area of contact and desired tightness of fit. Each AutoCAD design (.dwg format) was exported in .stl (stereolithography) format, which describes the surface geometry of a 3D object. To print, the .stl files were imported to Insight software for pre-processing and then relayed to Control Center software for printer communication.

A Stratasys Fortus 250mc was used to print all system components in acrylonitrile butadiene styrene (ABS) Plus 430 ($130/60in3 with educational discount). We strongly discourage the use of polylactic acid (PLA) material for 3D-printing any components of the system due to its low quality: it deforms with time and has a low melting point compared to ABS or ABSP. All parts were printed at high density at a layer thickness of 0.01in (0.254 mm). For parts with complex geometry, such as hollow regions, printers ejected a soluble support material (SR-30, $250/60in3) during the printing process. After printing, the support material was dissolved off of the plastic part in a heated, sonicated solution of concentrated sodium hydroxide (Soluble Concentrate P400-SC, WaterWorks). The part was then soaked in DI water for approximately 5 hours to drain any fluids remaining in the pores of the plastic. Each part was allowed ample drying time (~24 hours) prior to assembly. The printing software provided the volume (in3) of model and support material used for each print job, which was used to calculate the cost of each component (Supplementary Table 5).

### Additional Preparation of 3D-Printed Components

To prevent leakage, the porous surface of each tissue chamber was treated with a 100% acetone vapor bath. The chamber was fully submerged in acetone vapor (~1 inch above boiling surface) for 3–6 second bursts until the surface appeared shiny and smooth. After a drying time of 24 hours, the chamber was tested for leakage using DI water. If leakage was present, the waterproofing process was repeated.

To allow the securing of tissue in place, a 0.5cm-thick layer of PDMS gel (DC 184 SYLGARD 0.5KG 1.1LB KIT, Krayden) was added to the inner bottom surface of the upright bath. To make the PDMS gel, first, a 10:1 ratio of the elastomer and curing agent was mixed together using a tongue depressor attached to an electric mixer for 10 minutes. Next, the mixture was centrifuged for 5 minutes to remove bubbles. The mixture was then slowly poured into the upright bath and left at room temperature for 24 hours to dry.

The sideways bath features a 37 × 41.44 mm optical glass window (Stock #43-927, Edmund Optics) that was adhered using a 1:1 epoxy resin (Model #20945, Devcon) mixture after the adjustable paddle was attached. To fit the slot on the chamber and leave room for glue, the glass was cut 1/32” shorter than the slot on each side. After a drying time of 24 hours, the sideways bath was tested for leakage using DI water. If leakage was present, glue was re-applied accordingly.

### Software

Utilizing previously described methods for signal processing of optical mapping data^31^, RHYTHM 1.2 was developed using Matlab 2017 and is compatible with .gsh/.gsd and .rsh/.rsd file formats. The open-source platform includes the code and user manual to analyze voltage and calcium data simultaneously collected using the 3D-printed system.

### Tissue Preparations

The system was tested to optically map Langendorff-perfused mouse hearts and rat cardiac organotypic slice preparations. Briefly, mice were anesthetized using isoflurane vapors, hearts were excised following thoracotomy, and the aorta was cannulated as previously described^46^. Mouse heart studies utilized the sideways system orientation. For the rat heart slices, approximately 1.5 × 1.5 cm section from the LV was collected and sliced into 400 μm thick sections using a precision vibrating microtome (Campden Instruments), as previously described^47^. Rat cardiac slice studies utilized the upright system orientation.

### Optical Mapping

The mouse hearts (n = 4) and rat slice (n = 1) were optically mapped as previously described^46,48,49^. Briefly, tissue was perfused/superfused with Tyrodes’s solution (130 mM NaCl, 24 mM NaHCO3, 1.2 mM NaH2PO4, 4 mM KCL, 1 mM MgCl2, 5.6 mM Glucose, 1.8 mM CaCl2, 15 mM BDM, pH ~7.4 with carbogen bubbling) The tissue was stained with RH237, a voltage sensitive dye, and Rhod2-AM, a calcium indicator^46^. The tissue was then paced with 2 ms stimuli at 1.5x threshold of stimulation and a basic cycle length (BCL) of 150 ms. Dyes were excited by halogen light (UHP-Mic-LED-520, Prizmatix) that first passed through an excitation filter (ET500/40×, Chroma) before reflecting off of a dichroic mirror (T550LPXR-UF1, Chroma) towards the tissue. The emitted light was collected using a 1X lens (Part #10450028, Leica) and then split into the voltage and calcium signals by a second dichroic mirror (T630LPXR-UF1, Chroma). Each light path contained an emission filter (590 ± 33 nm for calcium and 690 ± 50 nm nm for voltage, ET590/33 m and ET690/50 m, Chroma) and was then recorded using a MiCam Ultima L-type CMOS camera with 100 × 100 pixel resolution. Temperature was continuously monitored during experiments using a temperature probe (Part #MLT1401 and #ML312, AD Instruments) and maintained at ~37 °C by varying the flow rate of the circulation of bath perfusate.

Prior to each experiment, the cameras were aligned by the manual adjustment of the adjustable optics holder and camera lifts to ensure the spatially accurate alignment of the signals (see Supplementary text and Supplementary Fig. 6). After each experiment, the tubing and tissue chamber were rinsed with dilute HCl (0.1 M).

### Statistical Analysis

All data are reported as mean ± standard error. Student’s t-tests were performed to detected significant difference between groups. P < 0.05 was reported as significant.

### Code Availability

The Matlab code developed to analyze optical mapping data and sample optical data is available under open-source MIT license at: (https://github.com/optocardiography).

## Electronic supplementary material

Supplementary Information

## Data Availability

The optical mapping and ECG data used for system demonstration are available from the corresponding author upon request. The 3D-printed hardware designs will be available in both drawing (.dwg) and printing (.stl) format at: (https://github.com/optocardiography).
