# Supplementary material for: Open-Source Multiparametric Optocardiography
Source: Sci Rep. 2019 Jan 24;9:721. doi: 10.1038/s41598-018-36809-y (PMC6346041; doi:10.1038/s41598-018-36809-y)
Supplement: Supplementary file 1 — Supplementary Information [file 41598_2018_36809_MOESM1_ESM.pdf]

Supplementary Materials for:

Open-Source Multiparametric Optocardiography

Brianna Cathey[#,1], Sofian Obaid[#,1], Alexander M. Zolotarev[2], Roman A. Pryamonosov[2], Roman A. Syunyaev[2], Sharon A. George*[,1], Igor R. Efimov*[,1]

1. Department of Biomedical Engineering, George Washington University, Washington DC 20052, USA
2. Laboratory of Human Physiology, Moscow Institute of Physics and Technology, Moscow Russia

[#] equal contribution

* To whom correspondence should be addressed: efimov@gwu.edu and sharonag@email.gwu.edu

SUPPLEMENTARY TEXT

Lab Jack

Each lab jack was assembled using the 6-step method illustrated in Figure 2a. Once assembled, supported components are secured using the assembly mechanism illustrated in Supplementary Figure 1. The weight limit of the lab jack is 30kg.

Lab Jack Assembly Instructions:

In step I., the left and right supports (perfusion or optical) were secured to the plate hinge with nuts (Supplementary Figure 2). Step I. was repeated three times to complete four sets of plate hinges with supports attached. In step II., one side of the lab jack was assembled first, with one plate hinge attached to the underside of the top plate and a second plate hinge to the bottom plate on the same side. The plate hinges were secured using lab jack twist locks. In step III., the mid-hinges (one threaded and one un-threaded) were secured to the supports with nuts. In step IV., the remaining two plate hinges were attached to the top and bottom lab jack plates. The other side of the mid-hinges were then secured to the supports with nuts. While at its lowest height and with the top and bottom plates parallel to each other, the screw was placed through the mid-hinges with the distal end going through the un-threaded mid-hinge first (step V.), reaching its correct position with the screw head positioned flat against the un-threaded mid-hinge (step VI.). Finally, the bottom plate of the lab jack was secured to the breadboard using M6 screws and washers.

Tilting Platform

The tilting platform (Figure 2b) was used to switch the optical components on the optical lab jack from sideways to upright imaging mode.

Tilting Platform Assembly Instructions:

First, the platform raise was attached to the optical lab jack top plate using the sliding rails. The platform raise was secured in the horizontal direction using stoppers (Supplementary Figure 3). Next, the plate mount was attached to the platform raise using sliding rails and positioned flush with the front edge of the platform raise. The upright plate was then attached to the plate mount using an axial rod (Supplementary Figure 4). With the upright plate at rest in the horizontal position, the excitation and emission filter cubes were secured onto the upright plate using the sliding rails and the filter cube twist locks. For sideways imaging, the upright plate was left at rest in the horizontal position. For upright imaging, the upright plate was rotated 90° to the upright position and held in place using the upright stabilizer, which slides onto the slots on the plate mount and the underside of the upright plate. A third stopper was placed at the edge of the upright plate closest to the rotational axis for further support of the optical components.

Hydraulic Lift

The hydraulic lifts were used to vertically support and spatially align the cameras in both orientations. Two 25-35cm range hydraulic lifts supported the cameras during sideways imaging, while one 44-54cm height range supported the second camera during upright imaging. The weight limit of the hydraulic lift in 15kg.

Hydraulic Lift Assembly Instructions:

The components of each hydraulic lift (Figure 2c) include two 60mL syringes (Cat# 13-689-8, Fisher Scientific), a short length of silicon tubing to fit the syringes (Item # EW-96400-14, Cole-Parmer), a stopcock (Cat# 120722, Radnoti), and the 3D-printed components. To assemble, first, the 3D-printed base was attached to the breadboard with M6 screws. Next, a short piece of tubing was attached to an empty syringe that was then placed into the 3D-printed base with the tubing going through a hole centered at the bottom of the 3D-printed base. The 3D-printed top was then glued with a 1:1 epoxy-resin mixture to the handle of this syringe. Next, the second syringe was filled with 60mL of water, attached to a stopcock, and then secured to the free end of the silicon tubing using the stopcock. This syringe was used to adjust the height of the hydraulic lift (Supplementary Figure 5). At the desired height, the stopcock was placed in the off position.

Focusing the Cameras to Infinity

The cameras were focused to infinity prior to signal alignment. With the optical components fully assembled, the projection lens sleeves were detached from the emission filter cube, leaving everything else intact. By rotating each focal adjuster to adjust the distance between each camera and its lens, the cameras were focused onto a distant object. Once each camera was focused onto the distant object, the detached components were re-attached to the emission filter cube using the projection lens sleeves. We recommend that the cameras be refocused each time the system is re-assembled.

Emission Filter Cube Dichroic Mirror Alignment

The dichroic mirror in the emission filter cube splits the voltage and calcium signals and directs the signals to the two cameras. To ensure spatial alignment (each camera has the same field of view) between the two cameras, the dichroic mirror angle and camera height was adjusted as needed before each experiment. This process should be performed after focusing each camera. We recommend that the dichroic mirror alignment be performed prior to each experiment for calibration and to ensure consistency between experiments.

Signal Alignment Instructions:

First, the MiCAM system was turned on and the MiCAM imaging software was opened. In the acquisition setup menu, the camera mode was set to 'Dual-Camera (Camera2: Horizontal mirror).' Next, the Focus Monitor was turned on. To align the image along one axis, the adjustable optics holder in which the dichroic mirror was placed was adjusted at the pivotal axis shown in blue in Figure 3. To align the image along the second axis, the hydraulic lifts were used to finely adjust the camera height. Once the cameras appeared to have the same field of view on the focus monitor display, the Dual Overlap function (Supplementary Figure 6, 4th column) overlaid the two images to allow for any further adjustment. To secure the placement of the adjustable optics holder when satisfied with the alignment, two clips were placed on either side along the rail inside the emission filter cube. Finally, the top piece of the adjustable optics holder set that houses the emission filters was placed into the emission filter cube.

The subtraction of the two images from Camera 1 and Camera 2 (Supplementary Figure 6 column 3) was used to quantify image alignment. The images from each camera were converted to binary values to do a direct comparison. White pixels represent a mismatch between the two images while black pixels represent a match. By comparing the ratio of black pixels to the total number of pixels in the image, we demonstrate 99.07% alignment (aligned, top) and 85.62% (unaligned, bottom).

List of Components
Supplementary Tables 1-3 in the Excel document display and describe the individual parts in each component category: stage, optical, and perfusion. Each row in each table represents one CAD file that can be found on the open-source platform. Duplicates of parts are indicated in parenthesis next to the part name.

Cost Analysis
Supplementary Tables 4-6 in the Excel document present a cost breakdown and cost comparisons of 3D-printed components with commercially available parts for the full dual camera tandem lens system.

Software Validation
Supplementary Table 7 displays the mean voltage and calcium parameter values calculated using Rhythm 1.2. The control values for mouse hearts in this table are also reported in Table 1 of the manuscript.

Additional Information: System Performance
ABS Plus plastic is resistant to corrosion, sagging, aging, fraying and warping under the conditions of use. It is not recommended to use other plastics such as ABS or PLA because these are less resistant to mechanical stress. PLA also has a lower melting point than ABS and ABSP causing tissue chambers made out of PLA to warp when exposed to warm perfusate. Build-up of dried perfusate may cause breakage of the lab jack twist locks when under stress, so

we recommend printing extra incase one needs to be replaced. While the ABS Plus plastic is not resistant to vibration, optical mapping experiments are best performed on an air table or steel breadboard that absorbs vibrations from the environment. The acetone vapor bath used to waterproof the tissue chambers will cause minimal warping of the plastic due to melting. This will cause the inlet and outlet holes for the superfusate to become slightly smaller in diameter, which is a consideration when modifying the chambers for different tubing sizes.

Additional Information: Modification of Parts

Stage Components – Possible modifications include adjusting the lengths of the lab jack supports or the base component of the hydraulic lift to accommodate different height requirements. Lab jack and tilting platform plates can also be modified to secure different sized attachments. The camera cage dimensions can also be edited to fit a different sized camera that can still be attached to the camera end sleeve using the twist lock mechanism.

Optical Components – The mirror and filters holders can be modified to secure mirrors and filters of different sizes and shapes. This can be done by modifying the dimensions of the slots in the optics holders. The lens holders (objective and projection) can also be modified in inner diameter to fit different sized lenses.

Perfusion Components – The tissue chambers can be scaled up or down to fit a different sized preparation. Further, if additional fields of view are desired, additional slots can be added for the placement of more optical windows. For larger tissue preparations, it is important to take extra caution when waterproofing due to the additional pressure on the plastic surface that accompanies increased bath volume. It may be necessary to thicken walls of chambers for larger preparations.

SUPPLEMENTARY FIGURES 1-6 and Table 7

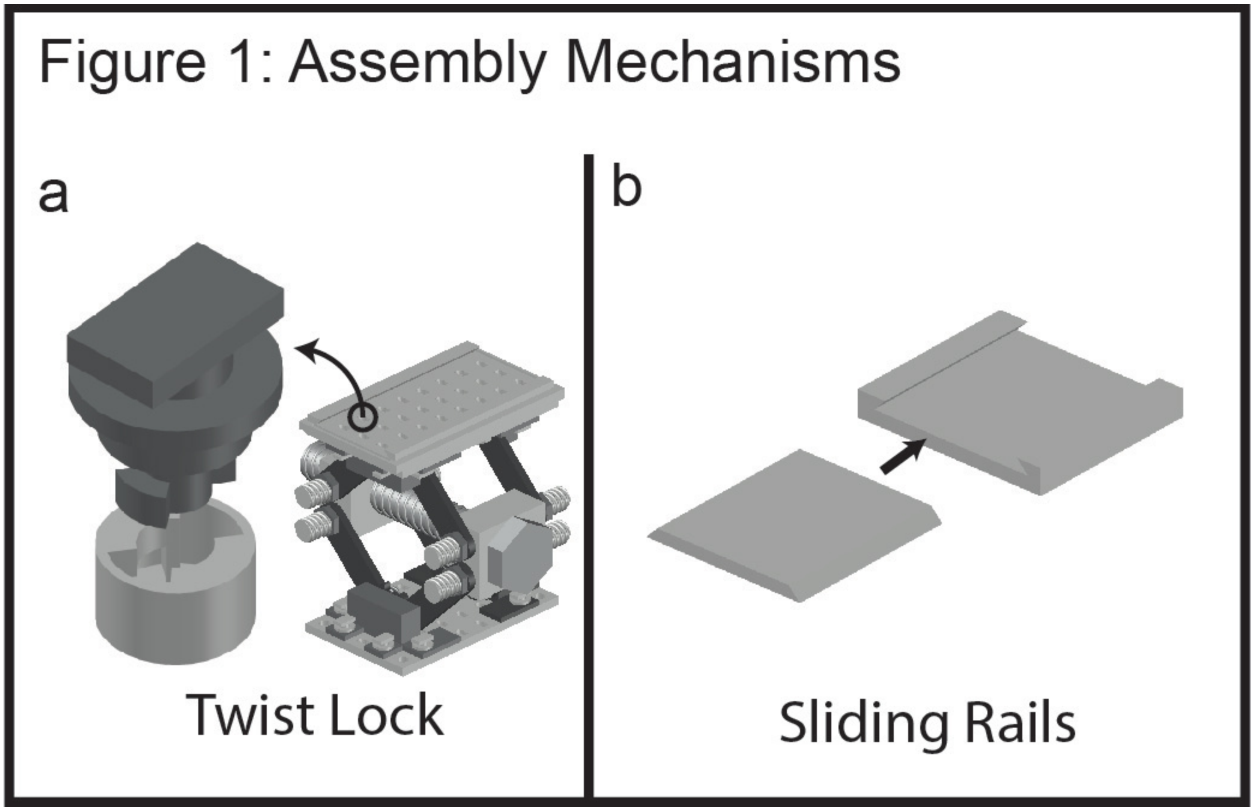

Figure 1: Twist lock **(a)** and sliding rail **(b)** mechanisms permit attachment of parts throughout system.

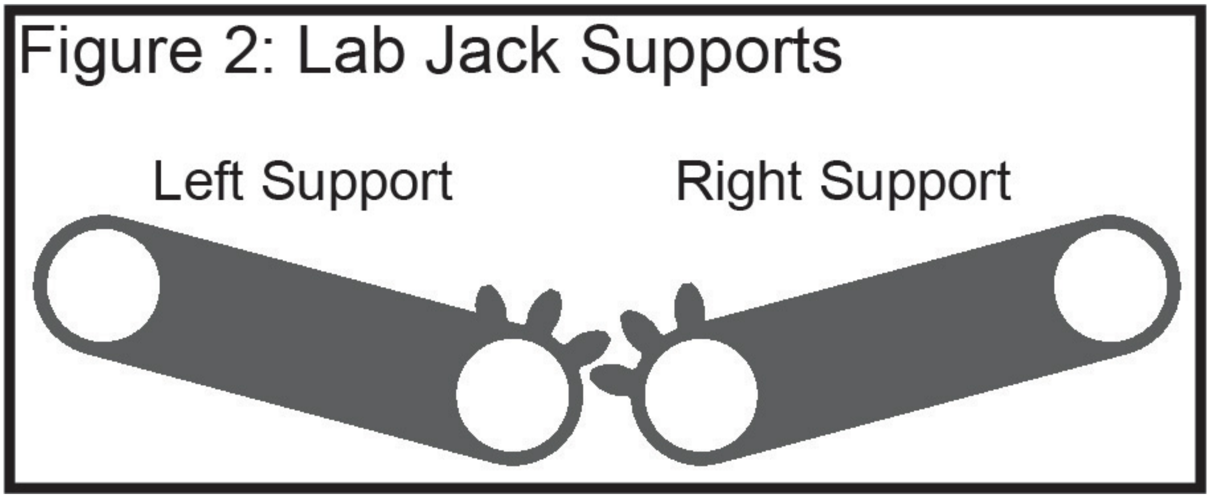

Figure 2: Left and Right Lab Jack Supports with gears

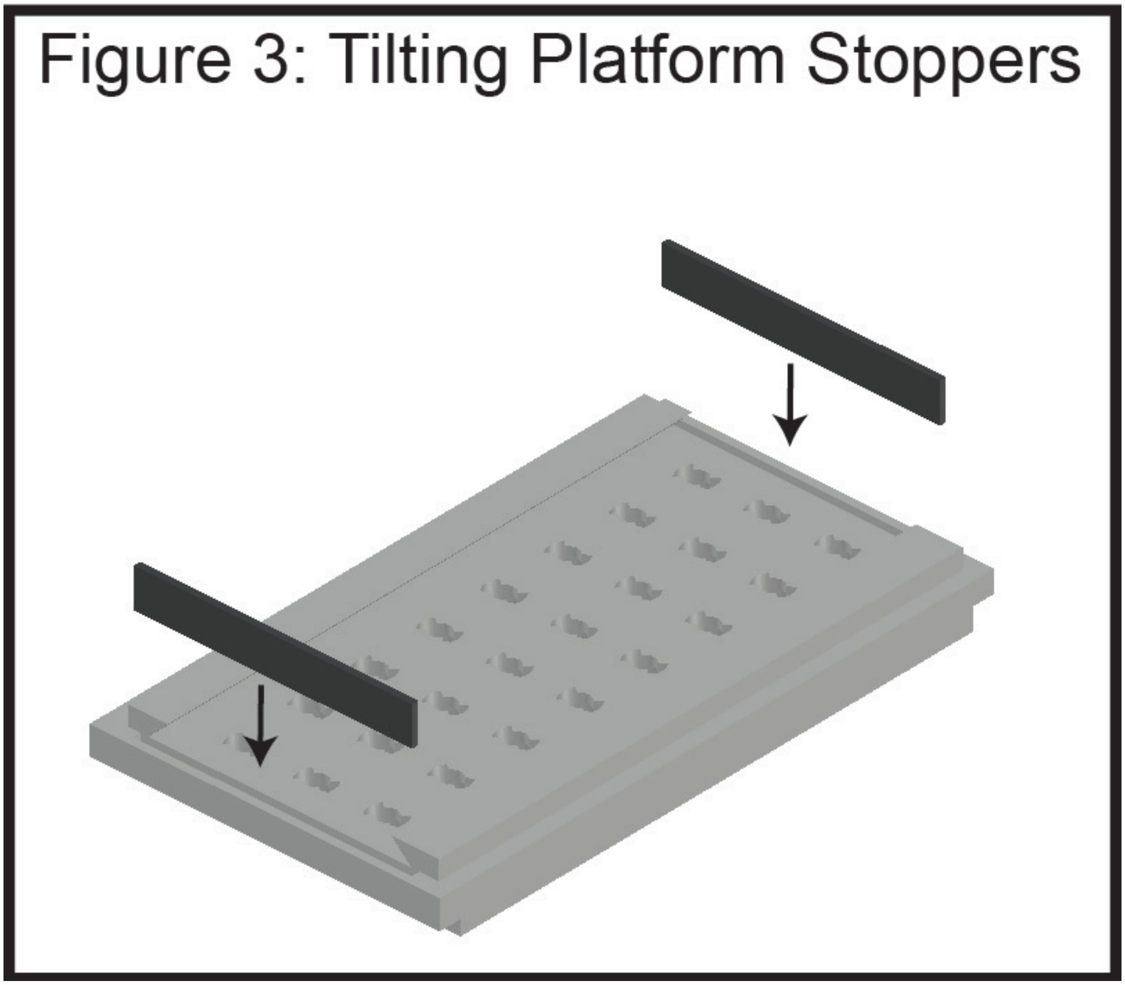

Figure 3: Stoppers (dark gray) placed in slots in top plate of optical lab jack to secure horizontal placement of the Platform Raise.

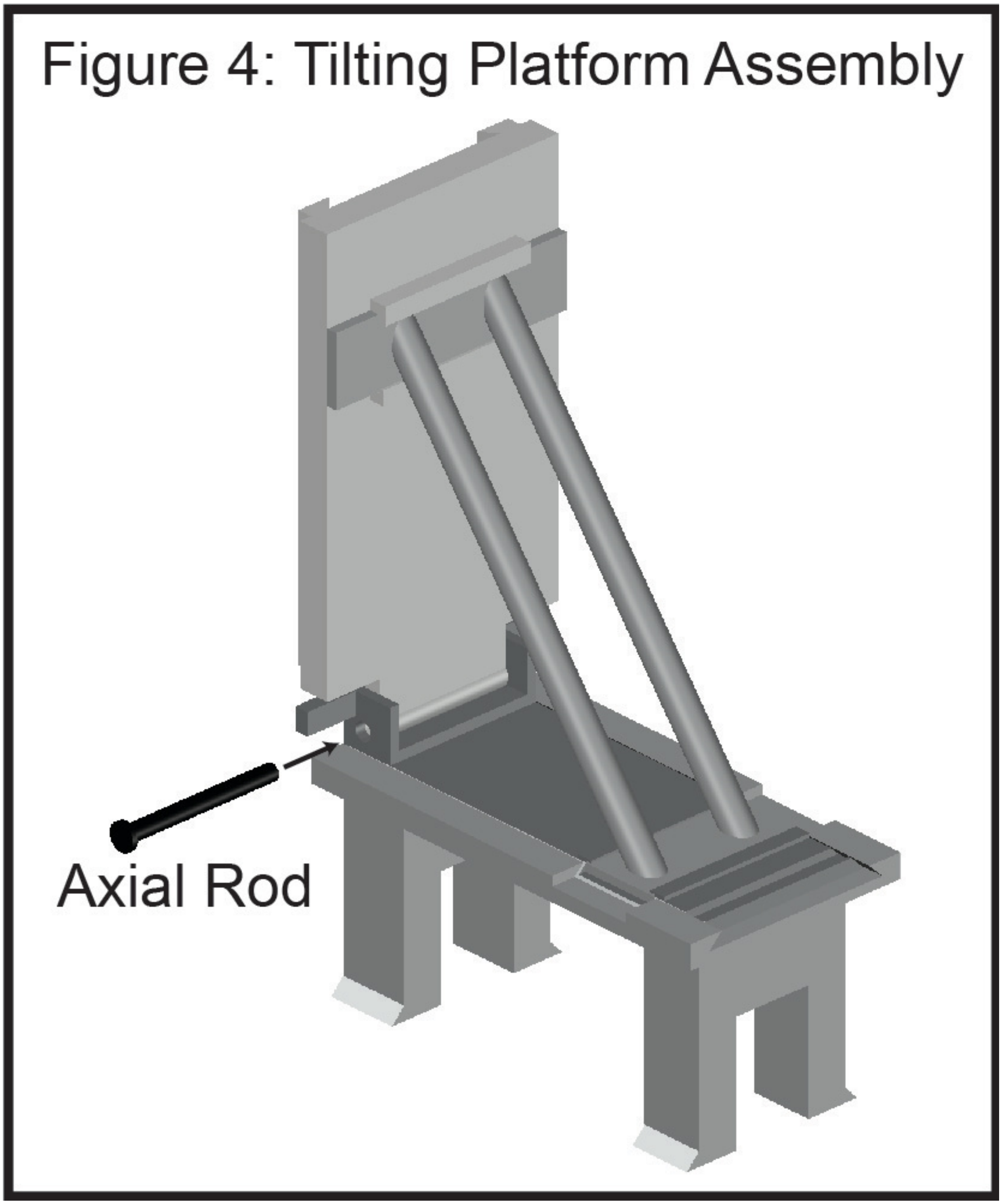

Figure 4: Axial Rod (black) secures Upright Plate to Upright Plate Mount to permit rotation of Upright Plate.

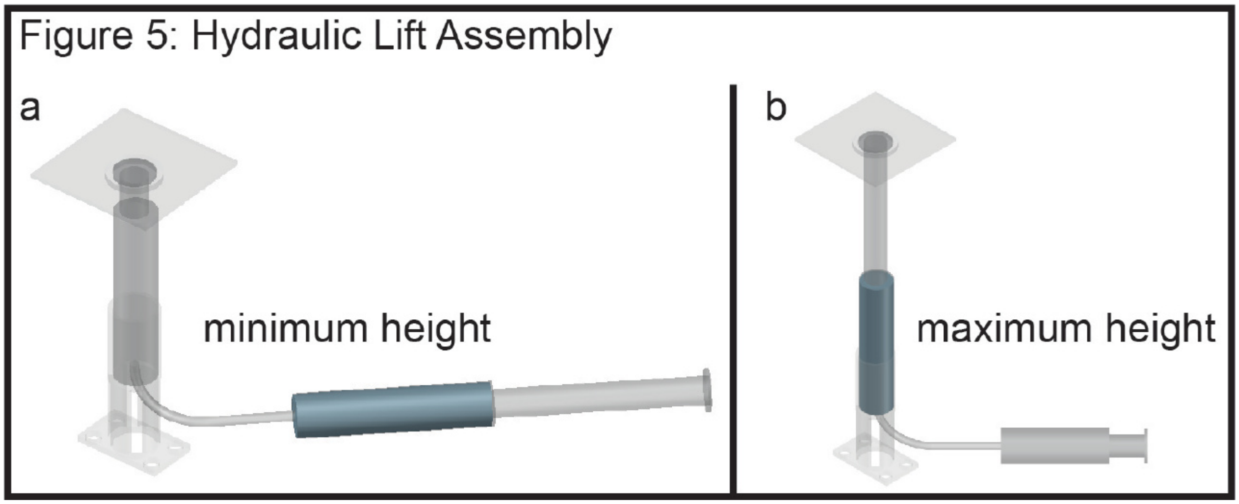

Figure 5: Hydraulic lift at minimum **(a)** and maximum **(b)** height. The water (blue) is displaced between two syringes connected by silicon tubing.

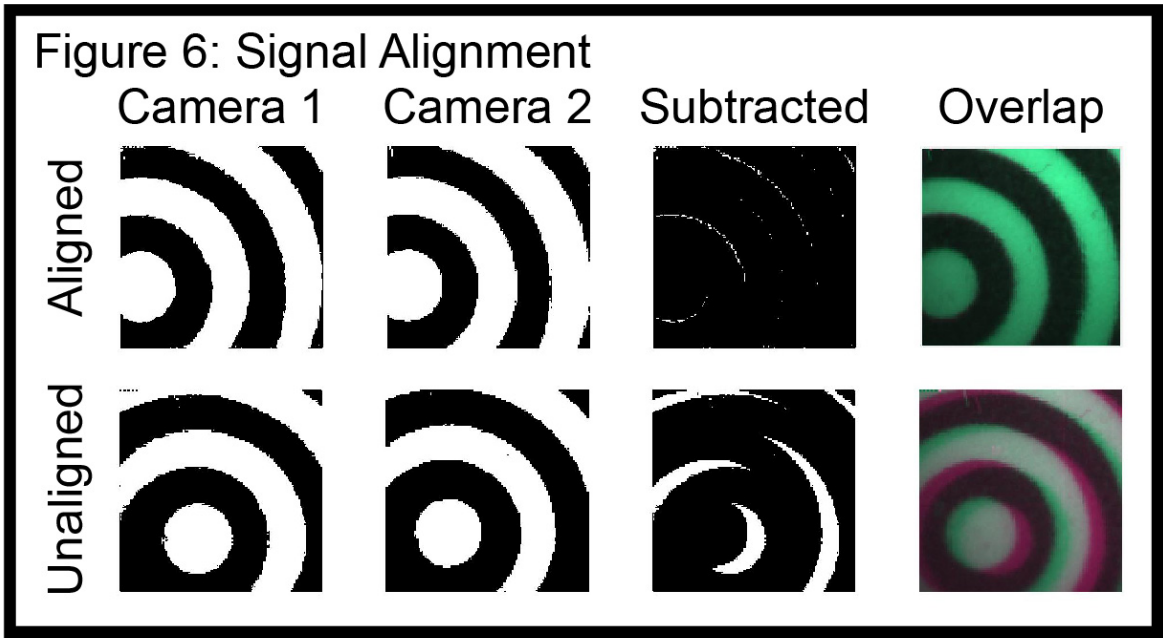

Figure 6: The aligned (top) and misaligned (bottom) views of Camera 1 and Camera 2. The 'Subtracted' column shows the binary subtraction of Camera 1 and Camera 2 views, where the black represents aligned pixels. The 'overlap' column displays the overlap of the two camera views using the Dual Overlap feature in the MiCAM BV_Ana software.

Table 7: Mean Parameter Values Calculated using Rhythm 1.2

	Mouse Heart (n=4)		Rat Slice (n=1)	
Parameter	Voltage	Calcium	Voltage	Calcium
Transverse CV (m/s)	0.30		0.23	
Longitudinal CV (m/s)	0.67			
Action Potential/Calcium Transient Duration (ms)	68.23 (APD80)	70.82 (CaTD80)	44.70 (APD80)	60.87 (CaTD80)
Rise Time (20-90%) (ms)	5.05	13.98	11.04	18.52
Calcium Decay Constant, Tau (s^{-1})		32.78		204.21

Supplementary Materials for:
Open-Source Multiparametric Optocardiography
Brianna Cathey[#,1], Sofian Obaid[#,1], Alexander M. Zolotarev[2], Roman A. Pryamonosov[2], Roman A. Syunyaev[2], Sharon A. George[*,1], Igor R. Efimov[*,1]
1. Department of Biomedical Engineering, George Washington University, Washington DC 20052, USA
2. Laboratory of Human Physiology, Moscow Institute of Physics and Technology, Moscow Russia
# equal contribution
* To whom correspondence should be addressed: efimov@gwu.edu and sharonag@email.gwu.edu

	Stage Components Parts List		
	Part Name/ Description	CAD drawing	Photo
Lab Jack	**Perfusion Lab Jack Supports (4L, 4R)**<br><br>Height range permitted: 27-34cm	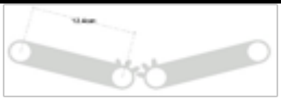	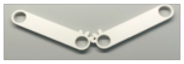
	**Optical Lab Jack Supports (4L, 4R)**<br><br>Height range permitted: 15-23cm	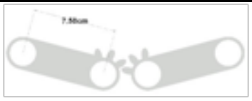	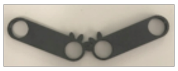
	**Plate Hinge (4)**<br><br>Provide pivotal axes for Lab Jack Supports.	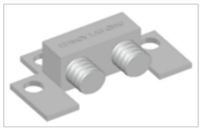	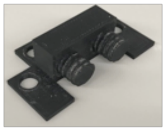
	**Top Plate**	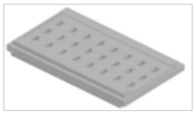	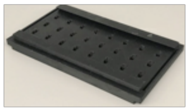
	**Bottom Plate**<br><br>Screwed onto breadboard. Fits metric or imperial breadboards.	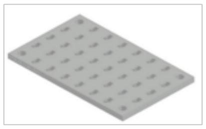	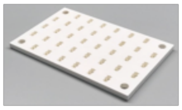
	**Threaded Mid-hinge**	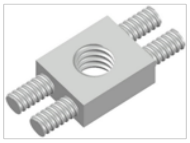	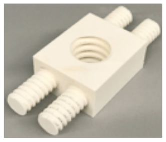
	**Un-threaded Mid-hinge**	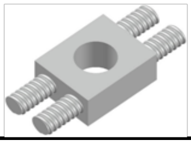	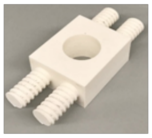
	**Nut (16)**<br><br>Secure Lab Jack Supports to the Plate Hinges and Mid-Hinges	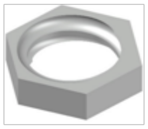	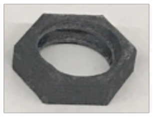
	**Lab Jack Twist Lock (16)**<br><br>Secure Plate Hinges to Top and Bottom Lab Jack Plate.	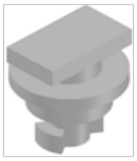	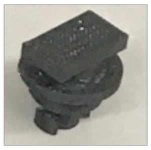
	**Lab Jack Screw**		

	Head of screw secured against Un-threaded Mid-Hinge	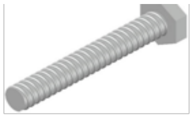	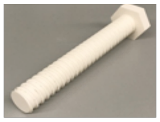
Tilting Platform	**Platform Raise**<br><br>Mounts directly onto Optical Lab Jack to provide option for upright imaging.	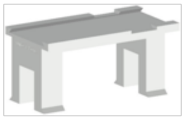	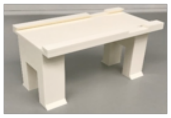
	**Upright Plate**<br><br>Secures optical components. Attached to the Plate Mount using the Axial Rod.	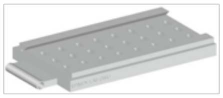	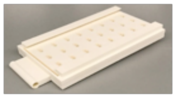
	**Upright Stabilizer**<br><br>Secures Upright Plate to the Plate Mount to hold optical components at a right angle.	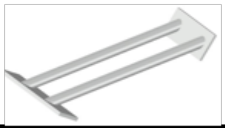	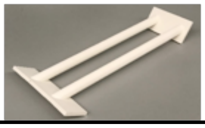
	**Upright Plate Mount**<br><br>Mounts directly onto Platform Raise.	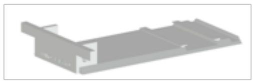	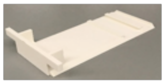
	**Axial Rod**<br><br>Secures Upright Plate to Upright Plate Mount. Provides axis of rotation for Upright Plate.	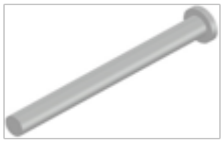	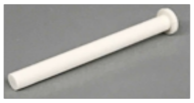
	**Stopper (3)**<br><br>Two are placed in slots on Optical Lab Jack on both ends of the Platform Raise. One is placed on the Tilting Platform in front of the Excitation Filter Cube when in upright imaging mode.	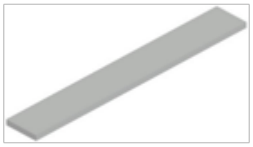	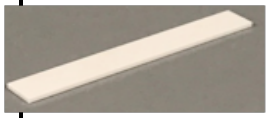
Lifts	**Upright Bath Lift (16-23cm)**<br>Provides vertical adjustment of Upright Bath. A screw secures the position of the lift. The base piece (left) is screwed onto the breadboard.	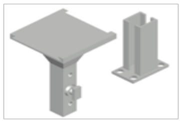	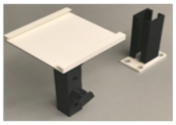
	**Hydraulic lift (2) (25-35cm)**<br><br>Supports Camera 1 and Camera 2 during sideways imaging.	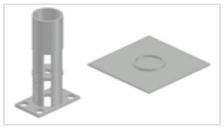	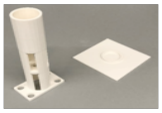
	**Hydraulic lift (44-54cm)**<br><br>Supports Camera 2 during upright imaging.	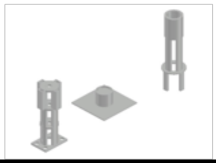	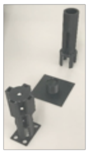
	**Camera Cage (2)**<br><br>Attaches camera to Camera End Sleeve.	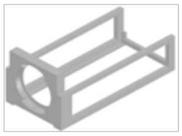	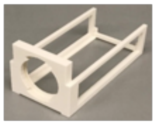

For questions, please contact: optocardiography@gmail.com

Optical Components Parts List		
Part Name/ Description	CAD drawing	Photo
**Objective Lens Sleeve Set**<br><br>The objective lens is placed as shown in the photo. The cap is then screwed on to secure lens placement.	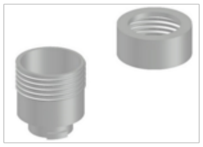	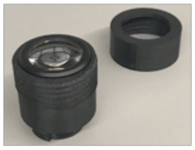
**Objective Lens Sleeve Cover**<br><br>Covers the objective lens when the system is not in use.	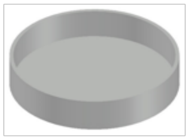	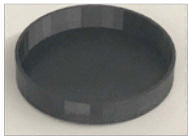
**Excitation Filter Cube**<br><br>Houses the Stationary Optics Holder. An excitation filter is placed in the small cylindrical slot (left).	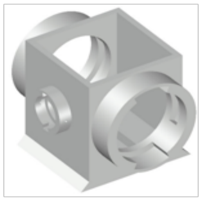	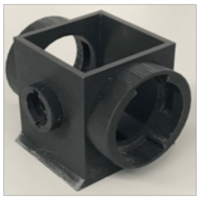
**Stationary Optics Holder**<br><br>Holds dichroic mirror in rectangular slot. For single-camera studies, an emission filter is placed in the circular slot. The dichroic mirror guides excitation light to tissue preparation and splits emission light from excitation light.	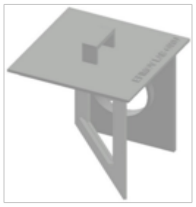	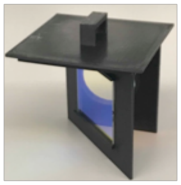
**Excitation Light Adaptor**<br><br>Secures excitation light guide to the Excitation Filter Cube	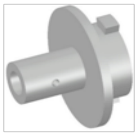	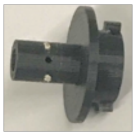
**Emission Filter Cube**<br><br>Houses Adjustable Wall and Emission Filter Holder.	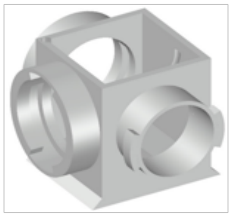	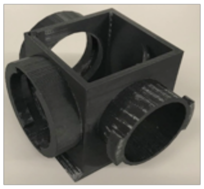
**Adjustable Wall**<br><br>Holds dichroic mirror that splits the voltage and calcium signals.	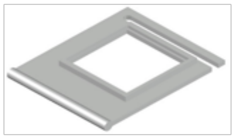	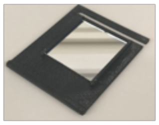
**Emission Filter Holder**<br><br>Holds emission filters that pass the wavelength of the emission spectra of each dye.	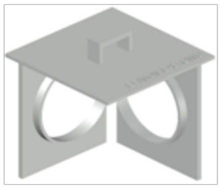	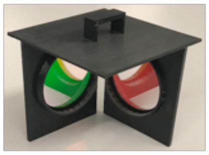
**Emission Filter Cube Clip(2)**		

Placed on curved rail inside Emission Filter Cube to hold Adjustable Wall in place.	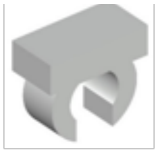	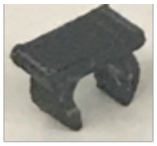
**Filter Cube Twist Locks (4) and Tool**<br><br>Secure Emission and Excitation Filter Cubes to Tilting Platform (or Optical Lab Jack).	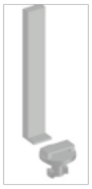	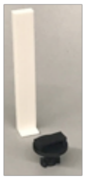
**Projection Lens Sleeve (2)**<br><br>Houses projection lens.	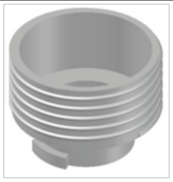	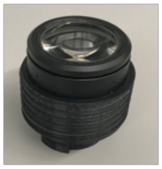
**Focal Adjuster (2)**<br><br>Adjusts the focus of projection lenses to infinity.	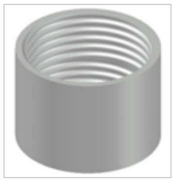	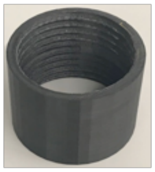
**Camera End Sleeve (2)**<br><br>Attaches camera to Focal Adjuster.	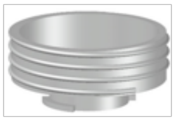	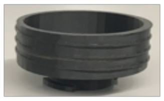
**Emission Filter Cube Cap**<br><br>Placed on Projection Lens attachment site when doing single-camera studies.	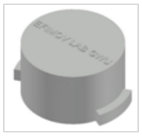	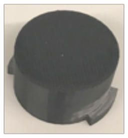

For questions, please contact: optocardiography@gmail.com

	Perfusion Components Parts List		
	Part Name/ Description	CAD drawing	Photo
Sideways Imaging	**Sideways Bath and Screw**<br><br>Houses whole-heart preparation for sideways imaging. Screw secures Electrode Paddle.	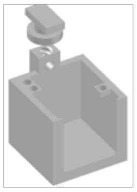	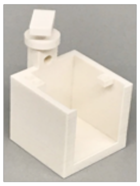
Sideways Imaging	**Electrode Paddle**<br><br>Placed in Sideways Bath. Holds pseudo-ECG electrodes and stabilizes heart against optical window of bath.	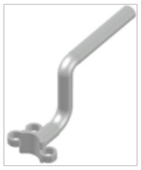	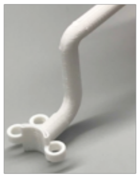
Sideways Imaging / Sideways Bath Stage	**Sideways Bath Linear Stage Mount**<br><br>Mounts the Sideways Bath Stage to Perfusion Lab Jack.	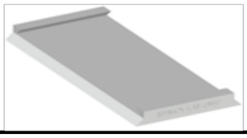	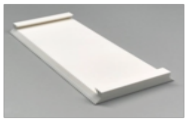
Sideways Imaging / Sideways Bath Stage	**Sideways bath Sliding Stage**<br><br>Houses Sideways Bath and Cannula Holder. Placed onto Sideways Bath Linear Stage Mount.	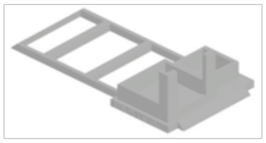	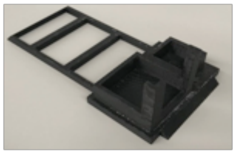
Sideways Imaging	**Cannula Holder Base**<br><br>Placed directly into slot on Sideways Bath Stage.	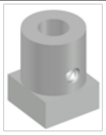	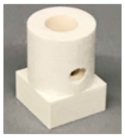
Sideways Imaging	**Cannula Holder Adjuster**<br><br>Attaches to Cannula Holder Rod and three-prong extension clamp. Permits vertical and horizontal adjustment of cannula.	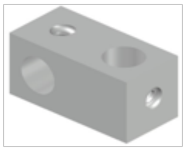	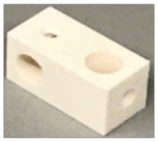
Sideways Imaging	**Cannula Holder Rod**<br><br>Placed into Cannula Holder Base. Permits vertical adjustment of cannula position.	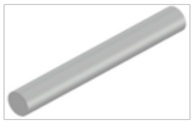	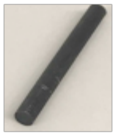
Sideways Imaging	**Cannula Holder Screw (3)**<br><br>Two screws secure the Cannula Holder Adjuster onto the Cannula Holder Rod and three-prong extension clamp. One screw secures the Cannula Holder Rod to the Cannula Holder Base.	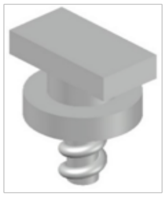	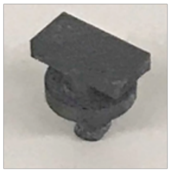
ight Imaging	**Upright Bath**<br><br>Houses tissue slice preparation or other preparations (e.g. atrial prep) for upright imaging.	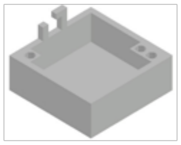	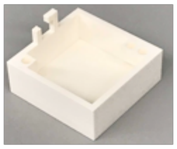
ight Imaging	**Upright Bath Stage**		

Upri	Houses Upright Bath and is mounted onto the Upright Bath Sliding Stage.	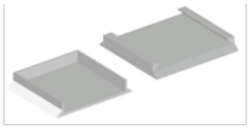	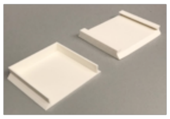

For questions, please contact: optocardiography@gmail.com

Cost Comparison: 3D-printed Components vs. Commercially Available Optomechanical Components			
**Components**	**Commercial Cost of Implementation ($)**	**3D-printed Cost of Implementation ($)**	**Cost Difference ($)**
**Optical Components**			
Excitation Light Adaptor	500	2.19	497.81
Excitation Filter Cube	8000	57.2	7942.8
Emission Filter Cube	11000	66.86	10933.14
**Perfusion Components**			
Vertical and Upright Bath	308.24	16.88	291.36
Linear Stages	682-1330	46.3	635.7
**Stage Components**			
Hydraulic Lifts	234	83.76	150.24
Upright Bath Lift	65	32.98	32.02
Tilting Platform	325-397	195.46	129.54
Lab Jacks	1219.92-1664	610.09	609.83
Total	22334.16	1111.72	**Potential Savings = 21222.44**

Note: Implementation costs were calculated based on the requirements of a dual-camera tandem lens system. The 3D-printed cost of implementation for each part is calculated in Table 5. Commercial cost of implementation for each part is calculated in Table 6. For commercial components with a range of cost options, as shown in Table 6, the least expensive option was used to calculate values in the 'Total' row (row 16). For questions, please contact: optocardiography@gmail.com

Cost of 3D-Printed Optomechanical Components

Perfusion Components

Part Name	Model (in^3)	Support (in^3)	Number	Model Cost ($)	Support Cost ($)	Total Cost of Parts ($)
Electrode Paddle w/ Screw	3.01	0.75	1	$6.52	$3.13	$9.65
Sideways Bath Linear Stage Mount	6.49	0.99	1	$14.06	$4.13	$18.19
Sideways Bath Sliding Stage	4.62	0.88	1	$10.01	$3.67	$13.68
Sideways Bath	2.11	0.64	1	$4.57	$2.67	$7.24
Upright Bath	3.24	0.58	1	$7.02	$2.42	$9.44
Upright Bath Holder	1.59	0.37	1	$3.45	$1.54	$4.99
Upright Bath Sliding Stage	3.27	0.46	1	$7.09	$1.92	$9.00
**TOTAL**	**24.33**	**4.67**		**$52.72**	**$19.46**	**$72.17**

Optical Components

**Emission Filter Cube**						
Emission Filter Cube	10.61	4.23	1	$22.99	$17.63	$40.61
Adjustable Wall	0.92	0.74	1	$1.99	$3.08	$5.08
Emission Filter Cube Cap	1.97	0.58	1	$4.27	$2.42	$6.69
Emission Filter Cube Clip	0.01	0.01	2	$0.04	$0.08	$0.13
Emission Filter Holder	2.18	2.01	1	$4.72	$8.38	$13.10
**Total**	**15.69**	**7.57**		**$34.02**	**$31.58**	**$65.60**
**Excitation Filter Cube**						
Excitation Filter Cube	10.24	3.82	1	$22.19	$15.92	$38.10
Light Adaptor	0.47	0.27	1	$1.02	$1.13	$2.14
Stationary Optics Holder	2.41	2.31	1	$5.22	$9.63	$14.85
Filter Cube Twist Lock	0.11	0.08	4	$0.95	$1.33	$2.29
Filter Cube Twist Lock Tool	0.27	0.07	1	$0.59	$0.29	$0.88
**Total**	**13.5**	**6.55**		**$29.97**	**$28.29**	**$58.26**
**Lens Sleeves**						
Objective Lens Sleeve	6.91	2.59	1	$14.97	$10.79	$25.76
Objective Lens Sleeve Cap	3.81	2.69	1	$8.26	$11.21	$19.46
Objective Lens Cover	2.33	0.43	1	$5.05	$1.79	$6.84
Focal Adjustor	8.01	5.75	2	$34.71	$47.92	$82.63
Projection Lens Sleeve	4.76	2.58	2	$20.63	$21.50	$42.13
Camera End Sleeve Master	3.34	1.51	1	$7.24	$6.29	$13.53
Camera End Sleeve Slave	3.35	1.51	1	$7.26	$6.29	$13.55
**Total**	**32.51**	**17.06**		**$98.11**	**$105.79**	**$203.90**
**TOTAL**	**61.7**	**31.18**		**$162.09**	**$165.67**	**$327.76**

Stage Components

**Camera Cage**	2.69	2.16	2	$11.66	$18.00	$29.66
**Total**	**2.69**	**2.16**	**2**	**$11.66**	**$18.00**	**$29.66**
**Tilting Platform**						
Axial Rod	0.27	0.13	1	$0.59	$0.54	$1.13
Stopper	0.23	0.1	3	$1.50	$1.25	$2.75
Plate Mount	6.97	1.35	1	$15.10	$5.63	$20.73
Plaform Raise	25.91	3.48	1	$56.14	$14.50	$70.64
Stabilizer	5.62	4.48	1	$12.18	$18.67	$30.84
Upright Plate	20.69	5.29	1	$44.83	$22.04	$66.87
**Total**	**59.69**	**14.83**		**$130.33**	**$62.63**	**$192.95**
**Hydraulic Lifts**						
25-35cm	4.57	1.36	2	$19.80	$11.33	$31.14
44-54cm	12.62	5.73	1	$27.34	$23.88	$51.22
**Total**	**17.19**	**7.09**		**$47.15**	**$35.21**	**$82.36**
**Upright Bath Lift**						

Upright Bath Lift	6.55	2.68	1	$14.19	$11.17	$25.36
Lift Screw	0.27	0.11	1	$0.59	$0.46	$1.04
Stabilizer	2.52	0.15	1	$5.46	$0.63	$6.09
**Total**	**9.34**	**2.94**		**$20.24**	**$12.25**	**$32.49**
**Lab Jacks**						
Bottom Plate	9.18	1.75	2	$39.78	$14.58	$54.36
Nut	0.11	0.09	32	$7.63	$12.00	$19.63
Plate Hinge	4.19	0.49	8	$72.63	$16.33	$88.96
Screw	14.13	3.21	2	$61.23	$26.75	$87.98
Top Plate	18.84	2.72	2	$81.64	$22.67	$104.31
Twist Lock	0.16	0.08	32	$11.09	$10.67	$21.76
Threaded Mid-Hinge	10.87	1.6	2	$47.10	$13.33	$60.44
Un-Threaded Mid Hinge	10.77	1.04	2	$46.67	$8.67	$55.34
Optical Jack Support	1.8	0.36	8	$31.20	$12.00	$43.20
Perfusion Jack Support	2.92	0.52	8	$50.61	$17.33	$67.95
**Total**	**72.97**	**11.86**		**$449.58**	**$154.33**	**$603.92**
**TOTAL**	**161.88**	**38.88**		**$658.95**	**$282.42**	**$941.37**
**Total Cost of System**						**$1,341.29**

Notes:
Total Cost (column G) = Model Cost
Model Cost (column E) is based on cost of model material ($130/60in^3); found here: http://store.amtekcompany.com/dimension-abs-plus-model-material-p430/
Support Cost (column F) is based on cost of model material ($250/60in^3); found here: http://store.amtekcompany.com/soluble-support-material-sr-30/
Number (column D) of each part is based on requirements for complete dual-camera tandem lens system

For questions, please contact: optocardiography@gmail.com

Cost of Commercial Optomechanical Components

Component	Manufacturer and Link	Cost per Unit ($)	Cost of Implementation ($)	Description	Comparison with 3D-printed component
Linear Stages	Newport: Ball-bearing Linear Stage	262	1048 (4 units)	Allows fine adjustment along single axis within a 25.4mm travel range. Four must be purchased for dual axis adjustment of both tissue chambers.	While the combination of our linear stage mount and sliding stage provide -x and -y axis adjustment, two of the Newport linear stages must be purchased to achieve this. Our sideways bath linear stage also provides a much wider range of adjustment in both the -x and -y direction (65mm). Furthermore, our design allows the permanent positioning of the cannula during adjustment.
	Precision 100 TPI Adjustment Screw, 25.4 mm, Small Knob Side Lock	30	120 (4 units)		
	Thor Labs: PT1B/M - 25 mm Translation Stage with 1/4"-170 Adjustment Screw M6 Taps	215.22	860.88 (4 units)	Allows fine adjustment along single axis within a 25mm travel range. Four must be purchased for dual axis adjustment of both tissue chambers.	(see above)
	Thor Labs: DTS25/M - 25 mm Dovetail Translation Stage M6 Taps	187.68	750.72 (4 units)	Allows single axis adjustment within 25mm range. Four must be purchased for dual axis adjustment of both tissue chambers.	(see above)
	Compact Dovetail Linear XY Stage, 25 mm Travel, 65 x 65 x 48 mm, M6	324	648 (2 units)	Allows dual axis adjustment within 25 mm range in both directions. Two must be purchased for dual axis adjustment of both tissue chambers.	Require additional adpators to house chambers and do not meet the 65mm range of our sideways bath linear stage mount and sliding stage.
	Removable Knob Set, Standard Adjustment Screw Quantity 3	17	34 (2 units)		
	Edmund Optics: X-Y Positioning Stage	665	1330 (2 units)	Closest alternative; Allows dual axis adjustment with 35mm range in both directions. Two must be purchased for dual axis adjustment of both tissue chambers.	(see above)
Tissue Baths	Radnotti: Preparatory Tissue Bath #158400	308.24	308.24	Water-jacketed; inner diameter=80mm, depth=20mm; can be used for both whole mouse heart and cardiac tissue slices	While this chamber can be used for both tissue preparation types, the chamber cannot be scaled to accommodate different preparations. Must also find a way to adjust chamber for field of view adjustment and secure in place. In addition, our sideways bath has optical glass to provide a smooth imaging surface of the whole heart, while this does not.
Tilting Platform	Barn Door: American Grip Camera Wedge Mount Plate 90 Degree	397	397	Closest alternative	While this product is made of metal and is therefore sturdier in supporting the optical components, it does not provide versatility in attaching equipment to it compared to our upright plate that features a grid of twist locks and sliding rails for positioning.
	Future Studio Equipment: Camera Wedge Plate 0-90 degrees	325	325	Metal tilting platform with grid of screw holes	Heavier duty than our tilting platform, but will require metal screws to attach components; screw hole grid dimensions must be compatible with the components being attached.
Hydraulic Camera Lifts	Fisher Scientific: Support stand	98	196 (2units)	The support stand can be used to hold a shelf support upon which the camera can rest. Two of each of these parts is necessary to support two cameras simultaneously.	The support stand provides a wide range of vertical adjustment like the hydraulic lift, however the hydraulic lift provides more user-friendly and fine adjustment, which is necessary to ensure that the cameras are well-aligned with the signal. While scissor jacks are also useful for finer, more controlled height adjustment, most do not meet the height requirements of our system and take up a significant amount of space on the breadboard.
	Thomas Scientific: Shelf Support	19	38 (2 units)		
Lab Jack	Newport: Lab Jack, 160 x 240 mm, 120 mm Range, 100 mm Min. Height, 500 N Load	1332	1664 (2 units)	Heavy duty lab jack with 500N load capacity, 160x250mm top plate, and height range of 100-220mm	Like the lab jack of our system, the top plate of these alternative lab jacks have attachment sites for supported components, but these compatible components are normally only available from the same manufacturer. While many of these options have a higher load capacity, our system does not require this high of a capacity to hold the equipment. The lab jack in our system supports up to 25kg, which is far heavier than the totality of components it supports in the system. The height range of these lab jacks also cannot be modified to have a wider or different height range like our lab jack can by adjusting the length of the supports.
	Newport: High Load Lab Jack, 6.5 x 9.5 inch, 300 lb. Load Capacity	1088	2176 (2 units)	1334N load capacity, 6.5x9.5in. top plate, and height range of 76.2-155.2mm	
	Newport: Lab Jack, 86 x 86 mm, 105 mm Range, 65 mm Minimum Height, 60 N Load	966	1932 (2 units)	Light duty with 60N load capacity, 86x86mm top plate, and height range of 65-170mm	
	Edmund Optics: 220mm x 160mm x 12mm Metric Lab Jack	825	1650 (2 units)	30kg load capacity, 220x160mm top plate, height range of 100-170mm	
	Newport: High Load Lab Jack, 4.375 x 6.5 inch, 200 lb. Load Capacity	762	1524 (2 units)	900N load capacity, 4.375x6.5in top plate, height range of 63.5-108mm	
	Thor Labs: L490/M - Heavy Duty Lab Jack - Metric	609.96	1219.2 (2 units)	34kg load capacity, 4x7in top plate, 57.4mm height range	
Upright Bath Lift	Southern Labware: Laboratory Support Jack, Aluminum, 6x6"	65	65	6x6" scissor jack with 60-265mm height range	Allows for finer adjustment of the upright bath compared to upright bath lift, but takes up significantly more space
Excitation Filter Cube	SciMedia: THT Sideways	8000	8000	8cm cube housing stationary dichroic mirrror	Functionally the same, but compatible components (mirror and filter holders) are only available from Sci Media; alternatively, you can purchase the individual pieces of filter cube components from Thor Labs at: https://www.thorlabs.com/newgrouppage9.cfm?objectgroup_id=3940. Both options significantly compromise flexibility, ease of assembly, and ease of system design
Emission Filter Cube	SciMedia: THT Sideways	~11,000	11,000	8cm cube housing adjutable dichroic mirror	(see above)
Exciation Light Adaptor	SciMedia: THT Sideways	500	500	Attaches light guide to excitation filter cube	Functionally the same

Note: Total system cost is not calculated here because the compatibility of a system utilizing these alternative components has not been tested. Instead, Table 4 provides a comparison with 3D-printed components to compute the potential total saving based on the individual components listed in this table. For questions, please contact: optocardiography@gmail.com

Note: Gray background indicates that parts must be purchased together

The components included in this list have the ability to perform the neccessary function for a dual-camera tandem-lens system. This sheet includes all of the necessary parts of the full system. The cost of implementation (column D) is calculated based on the number of each part necessary for a complete system. The comparison (column F) does not take cost into account, but rather functionality, ease of use, versatility, etc.
